# Dynamic Polarization of Rab11a Modulates Crb2a Localization and Impacts Signaling to Regulate Retinal Neurogenesis

**DOI:** 10.3389/fcell.2020.608112

**Published:** 2021-02-09

**Authors:** Brian S. Clark, Joel B. Miesfeld, Michael A. Flinn, Ross F. Collery, Brian A. Link

**Affiliations:** ^1^Department of Cell Biology, Neurobiology and Anatomy, Medical College of Wisconsin, Milwaukee, WI, United States; ^2^Department of Physiology, Medical College of Wisconsin, Milwaukee, WI, United States; ^3^Department of Ophthalmology and Visual Sciences, Medical College of Wisconsin Eye Institute, Milwaukee, WI, United States

**Keywords:** neurogenesis, Rab11, endocytosis, recycling endosome, crumbs, interkinetic nuclear migration

## Abstract

Interkinetic nuclear migration (IKNM) is the process in which pseudostratified epithelial nuclei oscillate from the apical to basal surface and in phase with the mitotic cycle. In the zebrafish retina, neuroepithelial retinal progenitor cells (RPCs) increase Notch activity with apical movement of the nuclei, and the depth of nuclear migration correlates with the probability that the next cell division will be neurogenic. This study focuses on the mechanisms underlying the relationships between IKNM, cell signaling, and neurogenesis. In particular, we have explored the role IKNM has on endosome biology within RPCs. Through genetic manipulation and live imaging in zebrafish, we find that early (Rab5-positive) and recycling (Rab11a-positive) endosomes polarize in a dynamic fashion within RPCs and with reference to nuclear position. Functional analyses suggest that dynamic polarization of recycling endosomes and their activity within the neuroepithelia modulates the subcellular localization of Crb2a, consequently affecting multiple signaling pathways that impact neurogenesis including Notch, Hippo, and Wnt activities. As nuclear migration is heterogenous and asynchronous among RPCs, Rab11a-affected signaling within the neuroepithelia is modulated in a differential manner, providing mechanistic insight to the correlation of IKNM and selection of RPCs to undergo neurogenesis.

## Introduction

The developing vertebrate retina forms from a pseudo-stratified layer of multipotent neuroepithelial progenitor cells competent to generate all of the major cell types (six neuronal, one glial) of the mature retina (Turner et al., 1990). Retinal progenitor cells (RPCs) initially proliferate to expand the progenitor population and subsequently switch to differentiating cell divisions—a process termed neurogenesis. Terminal divisions of RPCs occur in a stereotyped manner, providing an evolutionarily conserved, predictable birth order of retinal neurons and glia, which suggests that progenitors pass through a series of competence states where they gain and then lose capacity to generate specific cell types. This is supported by analyses of both the fly and vertebrate retinas (Li et al., 2013; Sato et al., 2013; Suzuki T. et al., 2013), where competence to generate discrete cell types is based on a dynamic and progressive temporal pattern of transcription factor expression. Additionally, clonal analyses of RPCs indicate that they can generate the proper array and proportions of retinal cell types even in clonal culture, supporting intrinsic mechanisms for regulation of neurogenic potential and cell-type fate decisions during retinal development (Cayouette et al., 2003, 2006; Slater et al., 2009; Gomes et al., 2011; He et al., 2012).

Despite this progress, we still lack comprehensive understanding of how intrinsic properties direct RPCs to exit the cell cycle and determine cell-type fate decisions on an autonomous basis. Most research into this question has focused on transcription factors essential for specification of individual retinal cell fates. For example, the expression of the proneural bHLH transcription factor Atoh7 (Ath5), which precedes the initial wave of retinal neurogenesis just prior to the terminal cell division of an RPC, drives neurogenic fates (Brown et al., 2001; Poggi et al., 2005; Brzezinski et al., 2012; Chiodini et al., 2013; Miesfeld et al., 2018a). In zebrafish, Atoh7 daughter cells yield one ganglion cell and either a post-mitotic photoreceptor, amacrine, or horizontal cell (Poggi et al., 2005). Which cell intrinsic mechanisms determine whether an RPC will remain proliferative or express Atoh7 and become neurogenic? Studies using frog and chick retinas suggest a negative feedback loop where Notch pathway activation and Atoh7 provide instructive signals for proliferation or cell cycle exit (Agathocleous et al., 2009). Recent data further suggest that Notch signaling activates *Hes* gene expression and can lengthen the cell cycle to allow the accumulation of higher levels of Atoh7, essential to ganglion cell genesis and cell cycle exit (Chiodini et al., 2013; Miesfeld et al., 2018b, 2020). While it is clear that the activity of these transcription factors is instructive for cell fate decisions, less is known about the mechanisms that link cellular features and signaling to the heterogeneity of transcription factor expression and activity within individual RPCs prior to cell fate commitment.

One cellular feature linked to neurogenesis is interkinetic nuclear migration (IKNM), the process where the nuclei of polarized epithelial cells oscillate in phase with the cell cycle, which is correlative with cell cycle exit in some neuronal compartments (Smart, 1972; Frade, 2002; Murciano et al., 2002; Tsai et al., 2005; Baye and Link, 2007; Xie et al., 2007; Miyata, 2008; Ge et al., 2010). Nuclear migrations are facilitated by both intrinsic cytoskeletal reorganization and motor activities, as well as through non-autonomous forces by neighboring cells (Del Bene et al., 2008; Norden et al., 2009; Schenk et al., 2009; Tsai et al., 2010; Kosodo et al., 2011). As such, aspects of IKNM, particularly the amplitude of the apical–basal movements, are variable and stochastic between cells (Leung et al., 2011; Barrasso et al., 2018). Consistent with an important role for nuclear migration, zebrafish RPCs that have deep basal nuclear oscillations are more likely to divide in a neurogenic mode (Baye and Link, 2007). These data contribute to the “nuclear residence hypothesis,” which suggested that the correlation of nuclear position and cell cycle exit arises from asymmetries in local signaling environments (Murciano et al., 2002; Baye and Link, 2007; Del Bene et al., 2008; Taverna and Huttner, 2010). In particular, differences in Notch signaling based on nuclear position have been observed in zebrafish neuroepithelial cells, such that Notch activity increases as the nucleus migrates apically (Murciano et al., 2002; Del Bene et al., 2008). Along with nuclear migration, cell shape, but not cell cycle length, is predictive of cell division mode and cell-type fate based on the computational analysis of clonal RPCs imaged with time-lapse microscopy (Cohen et al., 2010).

**Figure 1:**
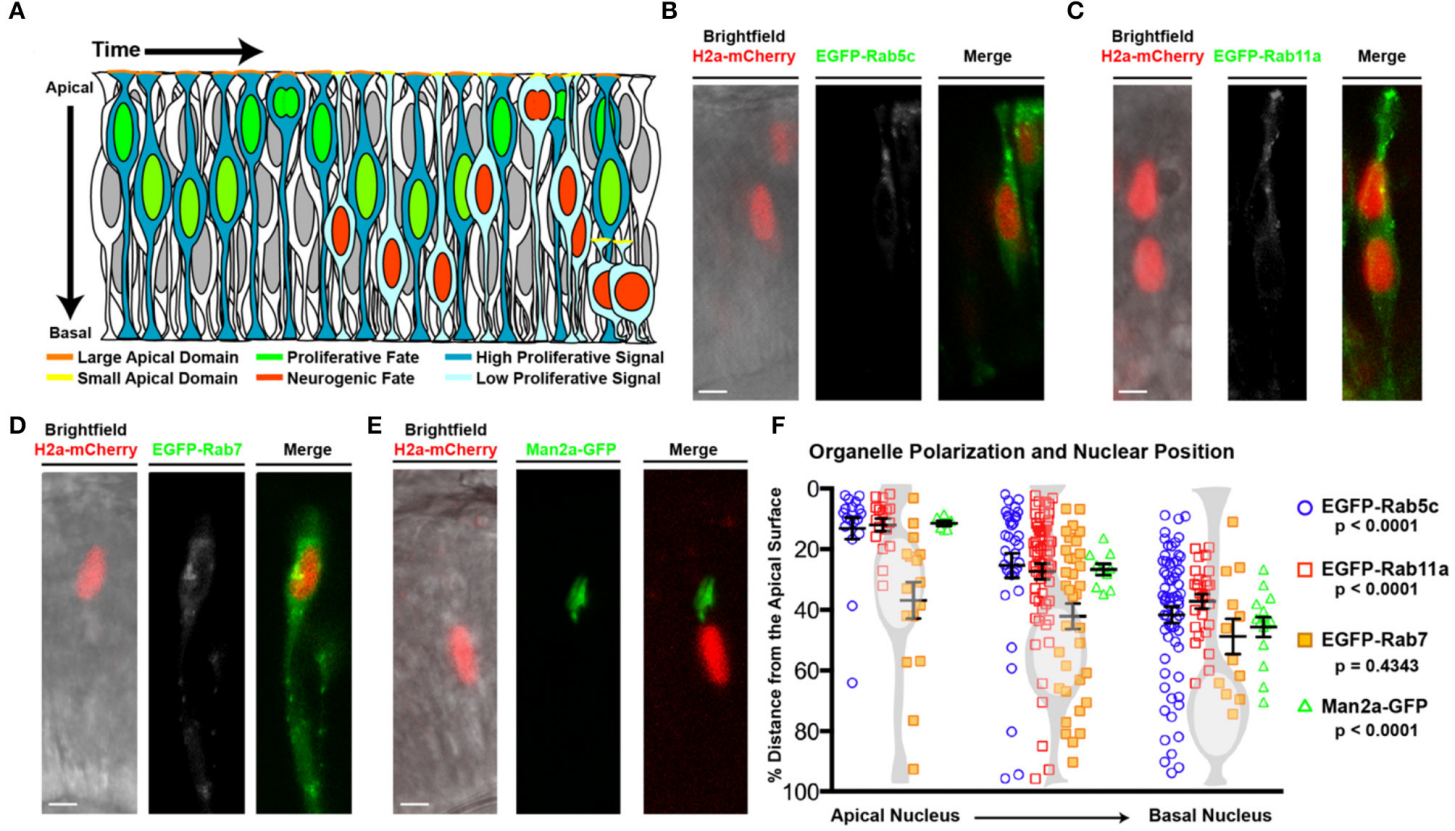
Organelle positioning during interkinetic nuclear migration. **(A)** Schematic of cellular features correlated with neurogenic and proliferative RPCs, including nuclear position, apical domain size, and proliferative signaling. **(B–E)** Examples of genetic mosaics of transplanted cells with H2a-mCherry labeled nuclei and endocytic organelles marked by EGFP-fusion proteins. **(B)** Early endosome (EGFP-Rab5c) localization in cells with apical nuclei. **(C)** Recycling endosome (EGFP-Rab11a) localization. **(D)** Late endosome (EGFP-Rab7) localization, and **(E)** localization of the medial Golgi apparatus (Man2a-GFP). **(F)** Quantification of the distance of organelles from the apical surface when nuclei are positioned apically (<25% of apical-basal distance), middle (25–50% of apical-basal distance), or basally (>50% of apical-basal distance). Data represent individual organelle positioning with mean and SEM indicated for each organelle type for each bin of nuclear positions from >10 cells/nuclear position bin from >5 embryos/genotype. Statistics represent results of a One-way ANOVA. Scale bars in **(B–E)** represent 10 μm.

The shape, polarity, and degree of connectivity of neural progenitors–established and maintained, in part, by the antagonistic functions of the Crumbs/Prkci/Par3/Par6 and Scribbled/Discs Large/Lgl complexes that facilitate apical–basal polarity, cell–cell junction formation, and preservation–are also important for cell fate outcomes (Cohen et al., 2010). For example, expansion of apical junctions and associated apical membrane autonomously increase Notch activity and maintain progenitors in a proliferative state (Clark et al., 2012). These observations and additional data on nuclear position and Notch signaling (Del Bene et al., 2008) suggest that both cell shape *via* apical junction remodeling and nuclear position *via* interphase oscillations impact signaling instructive for cell-fate decisions of RPCs (Figure 1A). The cellular mechanisms mediating the relationship between nuclear position, cell shape, and polarized signaling remain elusive, although, endocytosis may play a role (Nerli et al., 2020).

Endocytosis is the process by which proteins and lipids are trafficked in the vesicles between membrane-bound organelles and is a primary mechanism for how cell junctions are remodeled (Chalmers and Whitley, 2012). The members of the Rab family of small GTPases are effectors of endocytosis and specify the vesicle identity by functioning as molecular switches based on their GTP/GDP-bound state, which mediates the recruitment of effector proteins. Rab proteins and endocytosis regulate multiple cellular processes during development, including cell shape and polarity, in part through the maintenance of cellular junctions (Disanza et al., 2009; Orlando and Guo, 2009). Additionally, endocytosis can regulate signaling. The subcellular localization of endosomes can elicit polarized responses to form or modify morphogen gradients, whereas sorting of receptor–ligand complexes to distinct endosomal compartments can either prolong or quench signaling within cells (Lamaze and Prior, 2018). Specifically, in the retina, endocytosis was shown to modulate asymmetries in cellular signaling, including Notch signaling pathway activation, to control neurogenic decisions (Nerli et al., 2020). Based on these studies, we hypothesize that apical junction remodeling alters signaling in RPCs and biases neurogenic potential in a nuclear position-dependent manner. To gain further insight into the relationships between junction remodeling, polarized signaling, nuclear position, and neurogenesis, we examined the influence of localized endocytosis during retinal neurogenesis.

We previously generated and validated transgenic lines expressing fusions of wild-type (WT), dominant-negative (DN), and constitutive-active (CA) Rab proteins to facilitate our studies on the requirement of endosome biology on retinal neurogenesis and polarized signaling during *in vivo* zebrafish development (Clark et al., 2011). Using these lines, we show that the apical concentration of early and recycling endosomes in RPCs changes with respect to apical–basal nuclear position. To determine the potential consequence of polarized endosome concentration on retinal neurogenesis and polarized signaling within RPCs, we disrupted endosome recycling through the transgenic expression of the Rab11aDN. We demonstrate that Rab11a is required for proper localization of the apical junction protein Crumbs2a (Crb2a), and that changes to Rab11a function, Crb2a expression, and/or location alter retinal neurogenesis. Additionally, we show that changes in Rab11a activity *via* the Rab11a GTPase-activating protein (GAP) Evi5b promotes RPC proliferation. We provide evidence that several signaling pathways are modulated by localization of the Crb2a intracellular domain and affect cell cycle exit. Overall, these data suggest a model where variability in nuclear oscillations and apical endosome concentration alters polarized signaling among retinal progenitors, consequently promoting neuronal differentiation within the neuroepithelium.

## Results

### Determination of Organelle Polarity During Nuclear Oscillations

As nuclear position of RPCs correlates with both polarized signaling and cell cycle exit (Baye and Link, 2007; Del Bene et al., 2008), we examined whether localized endocytosis, known to regulate signaling in a variety of contexts, might mediate this relationship in RPCs. To investigate the positional relationship of endosomes with migrating nuclei, we first examined vesicle localization using transmission electron microscopy (TEM) images of 28 h post-fertilization (hpf) retinal neuroepithelial cells with either apical (within 5 μm distance to the apical surface) or more basal nuclei. We observed a higher concentration of vesicles within the apical zones of neuroepithelial cells when the nucleus was in close proximity to the apical domain; however, quantification using this method could not delineate endosome type (Supplementary Figures 1A,B). To determine the identity and relative concentration of individual endomembrane vesicles with respect to nuclear position, we utilized transgenic lines in which endosome sub-types are marked by GFP fusions of Rab protein isoforms. Chimeric embryos with isolated RPCs containing labeled endosomes (EGFP-Rabs) and nuclei (H2A-mCherry) were generated by blastulae transplantation of transgenic organelle-labeled cells into non-transgenic hosts (Supplementary Figure 1C). In neuroepithelial cells, both early (EGFP-Rab5c positive) and recycling (EGFP-Rab11a-positive) endosomes polarized toward the apical surface in a nuclear position-dependent manner (Figures 1B,C,F, Supplementary Figure 1D). The total number of marked endosomes was not significantly different between RPCs with different nuclear positions. To determine if nuclear position-dependent polarization is unique to these endosome sub-types, we examined other organelles including late endosomes (EGFP-Rab7; Figure 1D), centrosomes (Centrin-GFP; Supplementary Figures 1E,F), the *cis*-Golgi (Man2a-GFP; Figures 1E,F, Supplementary Figure 1G) and medial Golgi (Golga2-mCherry; Supplementary Figure 1K), mitochondria (CoxVIII-GFP; Supplementary Figures 1H–J), and endoplasmic reticulum (ER; DsRED-ER; Supplementary Figure 1L). The Golgi apparatus and ER both remained in close proximity to the nucleus, with the ER displaying perinuclear positioning and the Golgi moving relative to nuclear position, but always apical to the nucleus (Supplementary Figure 1G). However, the positioning of late endosomes (EGFP-Rab7), centrosomes (Centrin-GFP), and the mitochondrial network (CoxVIII-GFP) was not influenced by nuclear migration (Figure 1F, Supplementary Figures 1F,H–J). Combined, these data demonstrate the coordination of nuclear migration and polarization of the endomembrane secretory pathway including early endosomes, recycling endosomes, Golgi, and ER. We note that not all organelles exhibit nuclear position-dependent polarization. As a consequence of the endomembrane secretory pathway always apical of the nucleus (Ravichandran et al., 2020), we observe a significant concentration of the tubulovesicular pathway as the nucleus moves toward the apical surface. We hypothesize that the apical concentration of Rab5c- and Rab11a-positive early and recycling endosomes within RPCs facilitates junctional remodeling and polarized signaling, which could subsequently influence retinal neurogenesis.

### Rab11aDN Expression Alters Retinal Development, Expands Apical Junctions, and Redistributes Localization of Crb2a

To test the potential significance of dynamic endomembrane polarization on retinal signaling and neurogenesis, constitutive-active (CA) and dominant-negative (DN) isoforms of Rab proteins were utilized. Specifically, well-characterized mutant versions of Rab5c, Rab11a, and Rab7 that alter early, recycling, and late endosome activities, respectively, were expressed in the developing RPCs using the *vsx2*:Gal4;*UAS*:mCherry-Rab transgenes (Clark et al., 2011). The analysis of transgenic embryos revealed obvious retinal development defects in Rab11aDN eyes, including defects in retinal lamination and the presence of rosettes (Supplementary Figure 2). Only subtle abnormal phenotypes were observed in Rab5cCA embryos, and no defects were found in the other Rab mutant transgenic lines, due to either a true lack of function in retinal development, weak transgene expression, or functional redundancy by other Rab protein isoforms (Clark et al., 2011). Given the phenotypes associated with altered recycling endosome activity and the implication that Rab11 regulates cell junction integrity (Jing and Prekeris, 2009), we assessed the polarity of Rab11DN-expressing retinas by TEM and apical–basal junctional protein analysis.

**Figure 2:**
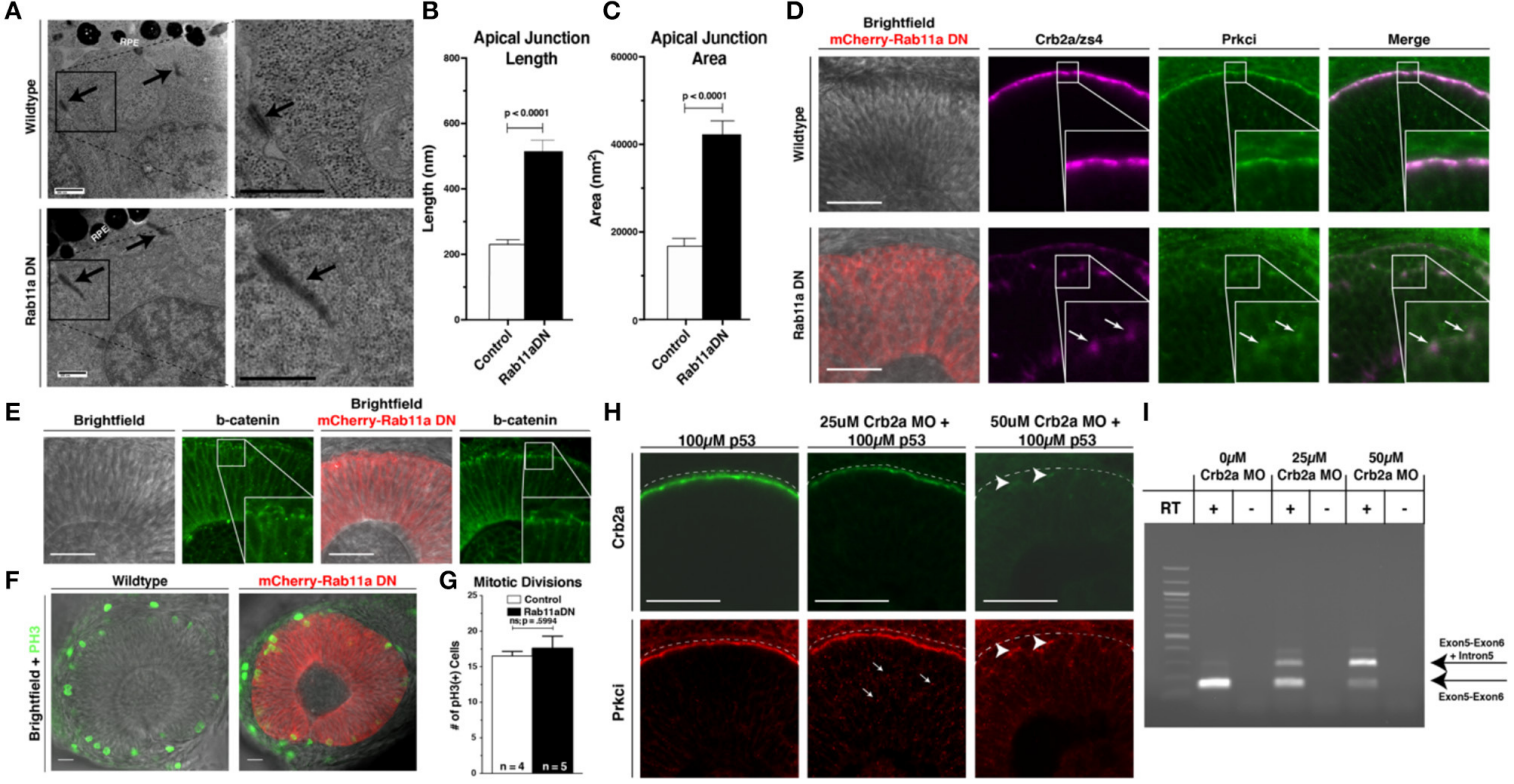
Rab11aDN expression causes Crb2a and Prkci mis-localization, but maintenance of additional features of apical-basal polarity. **(A)** Electron microscopy images of cells from 34 hpf Control (top panels) and Rab11aDN (bottom panels) retinas. Images are orientated at the apical surface, at the interface of the RPCs and Retinal Pigmented Epithelial (RPE) cells. Apical tight junctions are indicated by the black arrows. Black boxes (left panels) indicate higher magnification regions highlighted in the right panels. Scale bars = 500 nm. **(B,C)** Quantification of apical junction **(B)** length and **(C)** area from 34 hpf TEM images of Control and Rab11aDN-expressing RPCs. **(D)** Localization of Crb2a and Prkci within 28 hpf Control (top) and Rab11aDN (bottom) retinas. High magnification insets are outlined in the white squares. Arrows indicate ectopic localization of Crb2a and Prkci in Rab11aDN retinas. **(E)** Maintenance of the adherens junction protein ß-catenin localization in 28 hpf Control (left panels) and Rab11aDN (right panels) retinas. White squares indicate the regions of magnified insets. **(F)** Localization of mitoses as labeled by PH3 in Control (left) and Rab11aDN (right) retinas. **(G)** Quantification of the number of cells undergoing mitosis across the entire retina of 28 hpf Control and Rab11aDN retinas. Indicated n's represent total number of retinas quantified (1 retina/embryo). **(H)** Titration experiment of *crb2a* morpholino assessing the dosage-dependence of morpholino injection on Crb2a expression and Prkci localization. Increasing amounts of *crb2a* morpholino result in reduced Crb2a immunostaining and decreased accumulation of Prkci at the apical surface. Arrows indicate sites on non-apical Prkci localization in *crb2a* morphants, and arrowheads represent remaining Crb2a immunostaining accompanied by apical localization of Prkci staining. Dotted lines are positioned just above the apical surface (interface of RPE and progenitor cells). **(I)** Agarose gel of resulting PCR bands from RT-PCRs assessing efficiency of the splice-blocking *crb2a* morpholino within titration experiments. RT indicates the presence or absence of reverse transcriptase during cDNA synthesis. Arrows indicate the expect size of PCR products for the *crb2a* Exon5-6 junction (lower arrow) or across the *crb2a* Exon5-6 junction and including the *crb2a* intron 5. Scale bars in **(D–F,H)** represent 50 μm. Bar graphs represent mean values with error bars indicating standard error. Apical junction length and area **(B,C)** were quantified across 12 cells for each genotype, from 4 embryos/genotype. *P*-values represent statistical results of an unpaired *t*-test.

TEM studies revealed that overall polarity within Rab11aDN-expressing cells was maintained, including the presence of apically located cilia, even though histological sections showed developmental delay and lamination defects (Supplementary Figures 2, 3A,B). Both WT and Rab11aDN RPCs contained apical cilia even though previous studies have implicated that Rab11 plays a role in primary ciliogenesis of other cell types (Knodler et al., 2010; Westlake et al., 2011; Supplementary Figure 3A). However, we observed a notable change in the size of the apical junctions as a consequence of Rab11aDN expression (Figures 2A–C). In WT embryo TEM images, apical junctions appeared dense and compact (Figure 2A). Rab11aDN expression caused the apical junctions to appear more diffuse and expanded (Figures 2A–C), consistent with other manipulations that affect the localization of polarity proteins associated with junctions (Genevet et al., 2009; Hamaratoglu et al., 2009; Clark et al., 2012). We next asked if the morphological changes observed at the apical junctions correlate with changes to apical protein localization.

Further assessment of apical–basal polarity in Rab11aDN embryos was examined through the localization of the apical polarity proteins Crb2a and atypical protein kinase C (Prkci) (Figure 2D, Supplementary Figure 3C), adherens junction component beta-catenin (Figure 2E), and the apical F-actin belt (GFP-Utrph) and basement membrane protein Laminin-1 (Supplementary Figure 3D). Apical enrichment of beta-catenin and the F-actin marker GFP-Utrph and basal localization of Laminin-1 were all normal. In addition, mitotic divisions marked by phospho-histone H3 staining (PH3) occurred appropriately at the apical surface of the retinal neuroepithelium in both WT and Rab11aDN embryos (Figures 2F,G). Although overt polarity of Rab11aDN RPCs was maintained, we observed mis-localization of both Crb2a and Prkci away from the apical membrane at 28 and 48 hpf (Figure 2D, Supplementary Figure 3C), consistent with the altered localization of Crumbs proteins demonstrated in previous studies examining Rab11 loss-of-function conditions (Roeth et al., 2009; Schluter et al., 2009; Clark et al., 2011; Fletcher et al., 2012; Buckley et al., 2013). We also observed basal accumulation of Crb2a immunoreactivity and GFP-Utrph in Rab11aDN retinas, suggesting altered trafficking with loss of Rab11a function (arrows; Supplementary Figures 3C,D). In support of altered Crb2a trafficking through endosomes, Rab5cCA embryos also displayed Crb2a mis-localization, consistent with previous reports analyzing the internalization and recycling of Crumbs proteins at the apical domain (Supplementary Figure 3E; Lu and Bilder, 2005; Roeth et al., 2009; Clark et al., 2011; Fletcher et al., 2012).

Defects in endomembrane trafficking result in not only Crumbs mis-localization, as observed in our Rab11DN transgenics, but also degradation (Zhou et al., 2011). To measure the effects of Crb2a mis-localization and/or loss caused by Rab11aDN expression, we investigated the dosage dependence of Crb2a protein expression on Prkci localization using a splice-blocking Crb2a morpholino (MO) (Omori and Malicki, 2006). In WT control embryos, intense Crb2a staining was observed at the apical domain and Prkci mostly localized to the apical surface, with few intracellular puncta (Figures 2H,I). Injection of a sub-threshold dosage of Crb2a MO resulted in increased intracellular Prkci-positive puncta and a reduction in both apical Crb2a and Prkci staining (Figures 2H,I). Injection of a 50 μM Crb2a MO solution caused a complete loss of detectible Crb2a protein and minimal apical Prkci accumulation (Figures 2H,I). Comparisons of the Crb2a MO-injected embryos to the Rab11aDN embryos indicate that the Rab11aDN embryos display characteristics similar to a partial Crb2a loss-of-function, potentially through Crb2a mis-localization.

The analysis of polarity markers and retinal histology indicates that Rab11aDN expression resulted in altered Crb2a localization and diffuse junctions but maintained overt RPC apical–basal polarity. As we previously observed that expanded apical junction size led to decreased cell cycle exit (Clark et al., 2012), we sought to determine the extent to which altered Rab11a-dependent recycling affects RPC neurogenesis.

### Rab11aDN Expression Maintains RPC Proliferation

**Figure 3:**
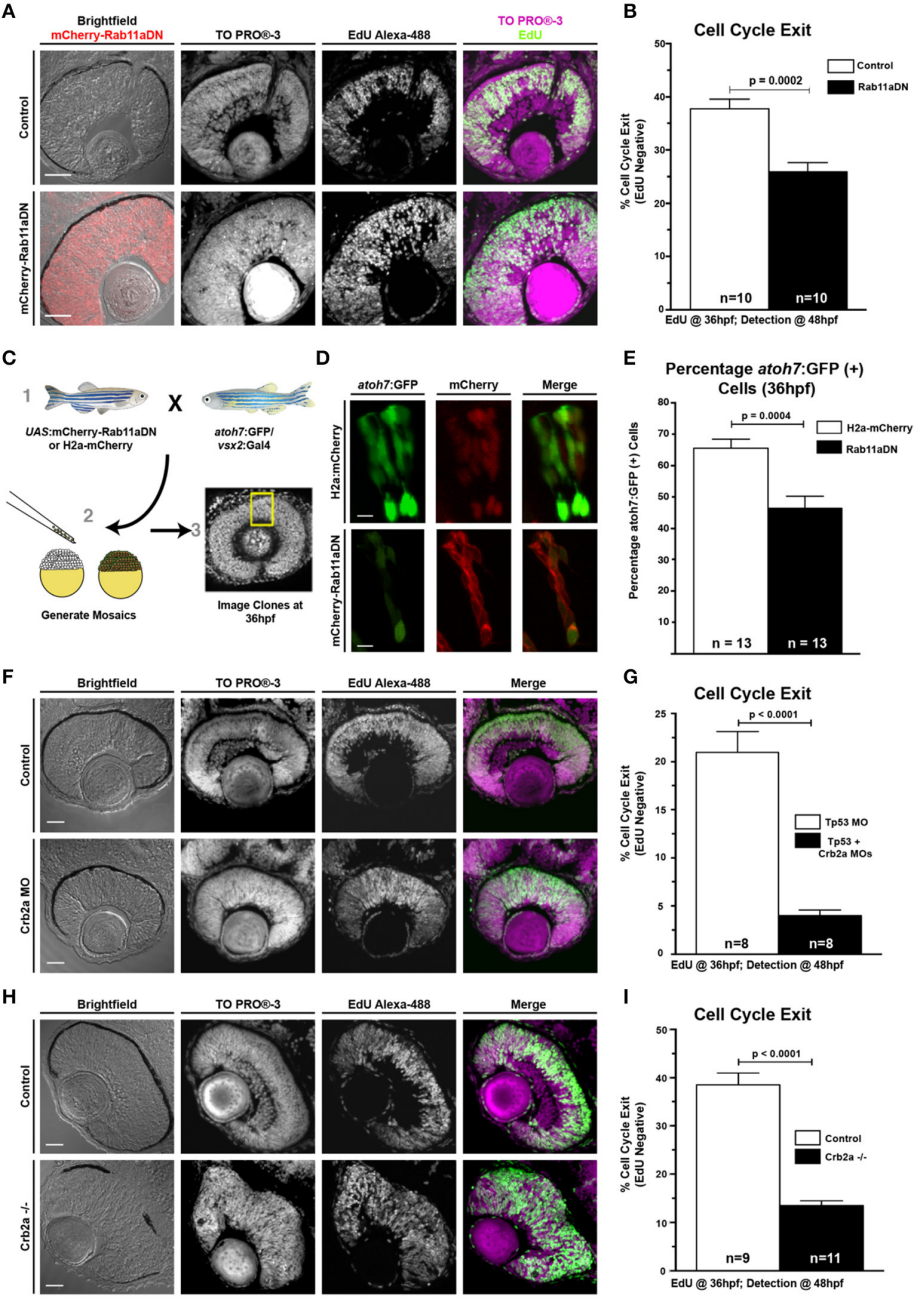
Rab11aDN expression and Crb2a loss-of-function promote RPC proliferation. **(A)** Representative retinal sections of Control (top) and Rab11aDN (bottom) retinas assessing RPC proliferation through detection of EdU incorporation at 48 hpf after a 12 h pulse from 36 to 48 hpf with nuclei counterstained with ToPRO-3. **(B)** Quantification of retinal cell cycle exit in Control and Rab11aDN EdU experiments. **(C)** Experimental design for assessing retinal neurogenesis in Rab11aDN genetic mosaics using the *atoh7*:GFP neurogenic reporter. **(D)** Representative images of Control (top) and Rab11aDN (bottom) genetic mosaics assessing retinal neurogenesis (*atoh7*:GFP). **(E)** Quantification of percentages of neurogenic cells (*atoh7*:GFP) in genetic mosaic experiments. Listed n's represent total number of clones assayed across >10 embryos/genotype. **(F,H)** Representative retinal sections of **(F)** Crb2a morphant or **(H)** Crb2a mutant retinas assessing RPC proliferation through detection of EdU incorporation at 48 hpf after a 12 h pulse from 36 to 48 hpf with nuclei counterstained with ToPRO-3. **(G,I)** Quantification of retinal cell cycle exit comparing Control and either **(G)** Crb2a morphant or **(I)** Crb2a mutant embryos. N's in **(G,I)** represent number of centrally localized retinal sections quantified, with 1 section counted/animal. Bar graphs in **(B,G,I)** represent mean with error bars indicating SEM. Statistics are the results of an unpaired *t*-test. Scale bars in **(A,C,F)** represent 50 μm, with the scale bars in **(D)** representing 15 μm.

Individual RPCs undergo neurogenic divisions in a nuclear position-dependent manner, suggesting that polarized cellular features, including the apical concentration of Rab11a-positive recycling endosomes, may influence RPC proliferation and differentiation. In addition, our data show that Rab11a function is essential for proper retinal histogenesis and localization of apical proteins. To determine how Rab11a function influences retinal neurogenesis, we analyzed the proportions of proliferative cells at 36 hpf, the end of the initial wave of retinal neurogenesis in zebrafish (Hu and Easter, 1999). To assess the proportion of cell cycle exit in Rab11aDN mutant retinas, we performed two separate experiments, with the first being an EdU incorporation study. EdU was injected into 36 hpf larvae and allowed to incorporate into cells progressing through S-phase over the course of a 12-h duration (36–48 hpf). This 12-h window is longer than the expected cell cycle of zebrafish RPCs (Baye and Link, 2007; Leung et al., 2011) and, therefore, provides a measure of the proportions of RPCs that exited the cell cycle within the 24–36 hpf developmental window prior to EdU treatment. The analysis of Rab11aDN retinas revealed a significant decrease in the percentage of EdU+ cells per retina indicating a reduction of RPC cell cycle exit (EdU-negative cells; Figures 3A,B). Second, to determine if Rab11aDN expression resulted in an autonomous decrease in cell cycle exit, we performed genetic mosaic experiments using donor embryos expressing the *atoh7*:GFP transgene, a marker that expresses in RPCs exiting the cell cycle during early retinal neurogenesis (Masai et al., 2003) and co-expressing either vsx2-driven H2a-mCherry or vsx2-driven mCherry-Rab11aDN. Neurogenesis was scored as the proportion of *atoh7*:GFP-positive cells within retinal clusters (GFP+mCherry+/mCherry+ cells) at 36 hpf (Figures 3C–E). In wild-type retinas, 65% of the transplanted cells expressed *atoh7*:GFP, whereas only 45% of the Rab11aDN cells were positive for *atoh7*:GFP expression, indicating that Rab11aDN expression caused an autonomous decrease in cell cycle exit (Figures 3D,E). Additional analysis at these early timepoints (24–28 hpf) indicated that there was no difference in the number of Rab11aDN vs. control progenitor cells in M-phase (Figures 2F,G) or undergoing cell death (Supplementary Figures 3F,G), suggesting that Rab11aDN expression biases RPCs to remain proliferative. However, increased cell death was observed at later timepoints (48–72 hpf), complicating interpretations of cell cycle exit of late progenitors as development progresses (Supplementary Figures 3F,G).

Because Rab11aDN results in Crb2a mis-localization and reduction at the apical surface, we assessed if Crb2a abundance was linked to the changes in Rab11aDN neurogenesis (Figures 3F–I). Both a partial reduction in Crb2a levels through MO injections or a complete loss of Crb2a protein (*crb2a* mutants) (Malicki and Driever, 1999) caused a significant decrease in the percentage of EdU+ cells per retina compared with controls. These decreases in cell cycle exit were similar to Rab11aDN retinas, suggesting that the neurogenic phenotype caused by Rab11aDN expression may, at least in part, result from improper trafficking affecting Crb2a abundance and/or localization.

### Rab11a Manipulations Do Not Affect Apical Domain Size

Changes to apical domain size can be caused by disruptions to several apical–basolateral polarity proteins including Crumbs family members (Omori and Malicki, 2006; Hsu and Jensen, 2010; Richardson and Pichaud, 2010) and Llgl1 (Clark et al., 2012). In the case of Llgl1, reduced protein expression in morphant embryos expanded the apical domain of RPCs, which resulted in decreased cell cycle exit due to increased Notch signaling (Clark et al., 2012). We therefore analyzed the apical domain area in Rab11aDN embryos using a retina-specific driven reporter of apical actin, *fzd5*:GFP-Utrh (Supplementary Figures 4A,B). Although Crb2a localization was altered in Rab11aDN embryos, the apical domain size of GFP-Rab11aDN-expressing RPCs was unaffected (Supplementary Figures 4C,D). These results suggest that the effects of Rab11DN expression on cell cycle exit are not due to altered apical domain size. However, Crb2a abundance or localization may have a more direct role on cell signaling that could affect neurogenesis. Indeed, distinct domains of Crumbs family proteins have been shown to regulate different cellular processes including cell signaling.

### Crb2a Regulates Cell Cycle Exit of RPCs

**Figure 4:**
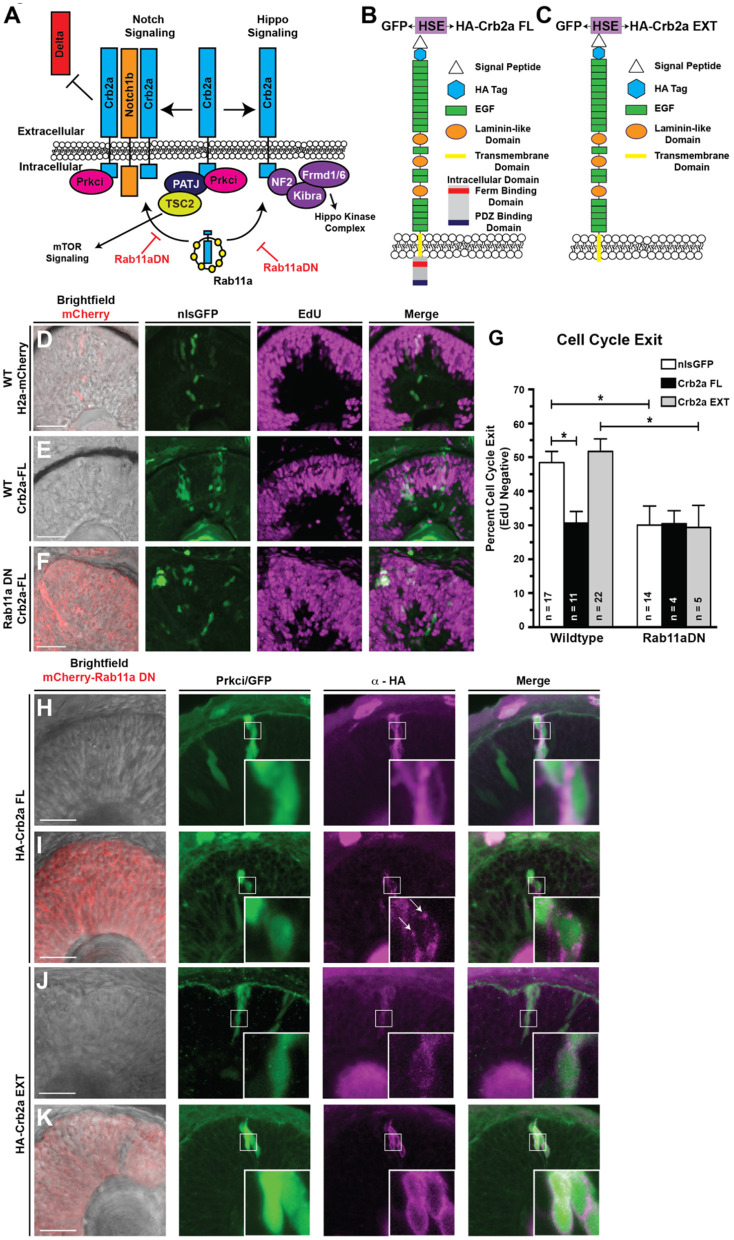
Ectopic expression of the full-length Crb2a promotes RPC proliferation. **(A)** Schematic of Crb2a function in the regulation of multiple signaling pathways. **(B,C)** Schematic of the **(B)** Crb2aFL and **(C)** Crb2aEXT transgene protein structures. HSE indicates the presence of an 8× repeat of the bi-directional heat-shock element to drive GFP and transgene expression simultaneously. **(D–F)** Representative images assessing levels of EdU incorporation within transgene clones (GFP), induced with a 30-min heat-shock at 24 hpf, after a 12-h EdU pulse from 36 to 48 hpf in **(D)** Control (WT background; H2a-mCherry; nlsGFP), **(E)** Crb2aFL (WT background; Crb2aFL) overexpression, or **(F)** Rab11aDN/Crb2aFL (Rab11aDN background; Crb2aFL) retinas. **(G)** Quantification of the percentages of cell cycle exit (EdU negative) within retinal sections. Listed n-values indicate the number of individual embryos counted for each genotype. Data represent the mean percent of cell cycle exit across clones, with error bars indicating SEM. * Indicate *p* < 0.05 after Tukey's multiple comparisons tests of a One-way ANOVA (*p* = 0.0002). **(H–K)** Representative images (>3 embryos assessed/genotype) of transgene overexpression of **(H,I)** Crb2aFL or **(J,K)** Crb2aEXT in either **(H,J)** WT or **(I,K)** Rab11aDN backgrounds, assessing localization of Crb2a transgene expression (HA tag). White boxes represent regions of highlighted in high magnification insets. Arrows in panel I represent Crb2aFL accumulation in puncta, suggesting accumulation of the transgene within non-plasma membrane associated focal puncta when expressed in the Rab11aDN background. Scale bars represent 50 μm.

To address the role of Crb2a and its different domains as part of the Rab11aDN phenotype, we generated several domain deletion transgenes. Crb2a is a single-pass transmembrane protein that contains a large extracellular domain with multiple EGF repeats and a short cytosolic domain that facilitates protein–protein interactions through FERM-binding and PDZ domains with proteins including Prkci (Bulgakova and Knust, 2009; Figure 4A). The extracellular domain is able to inhibit Notch signaling by binding the Notch receptor extracellular domain in *cis*, thus inhibiting the ligand activation of the receptor (Ohata et al., 2011). Reports also indicate that Crb regulates the Hippo and mammalian target of rapamycin (mTOR1) pathways through protein interactions of the Crb intracellular domain (Massey-Harroche et al., 2007; Genevet et al., 2009; Hamaratoglu et al., 2009; Chen et al., 2010; Grzeschik et al., 2010; Ling et al., 2010). As endocytosis of Crb2a would affect the availability of different domains for protein interactions, we developed several different Crb2a transgenes to address the potential function of each domain in regulating retinal neurogenesis (Figures 4B,C).

To first verify the expression and predicted functionality of our Crb2a overexpression transgenes, we examined the ability of each to rescue Prkci localization in Crb2a morphant backgrounds (Supplementary Figures 4E–J). Embryos carrying heat-shock inducible Crb2aFL (full length), Crb2aEXT (a Crb2a transgene lacking the intracellular domain), or GFP (as a control) were injected with a dose of Crb2a MO that eliminates apical Prkci localization (Figures 2H,I). We then performed genetic mosaics in which we induced clonal transgene expression through heat-shock at 24 hpf and subsequently analyzed Prkci immunostaining at 36 hpf. Predictably, the expression of the GFP or Crb2aEXT transgenes failed to localize Prkci to the apical surface due to the absence of the Prkci-binding domain normally located within the deleted Crb2a intracellular region. As expected, the Crb2aFL transgene was able to recover the apical localization of Prkci (Supplementary Figure 4J). We attempted to perform similar experiments using a Crb2aINT (Crb2a intracellular + transmembrane domain protein) transgene; however, we were unable to detect the presence of Crb2aINT protein when ectopically induced. We attribute this to the rapid degradation of the truncated Crb2a protein as both the *ha-crb2aINT* mRNA and the bi-directionally expressed GFP protein were robustly detected when the transgene was induced (data not shown).

Following the expression and functionality controls, we next tested whether the Crb2a transgenes could rescue cell cycle exit in Rab11aDN-expressing RPCs. Specifically, we assessed EdU incorporation from 36 to 48 hpf in cells where Crb2a isoforms were overexpressed through heat-shock activation at 24 hpf (GFP:HSE:HA-Crb2aFL/EXT). The expression of the Crb2aFL drove RPC proliferation, consistent with reports from *Drosophila* (Figures 4D–G) (Chen et al., 2010; Ling et al., 2010; Richardson and Pichaud, 2010; Robinson et al., 2010). However, the expression of the Crb2aEXT did not result in any change in RPC cell cycle exit compared with control retinas (Figure 4G). Interestingly, neither Crb2aFL nor Crb2aEXT isoforms were able to attenuate the reduced cell cycle exit caused by Rab11aDN expression (Figures 4F,G). Based on these results, we assessed the localization of overexpressed Crb2a (FL and EXT) protein in both WT and Rab11aDN embryos (Figures 4H–K). In wild-type cells, the overexpression of both Crb2a isoforms resulted in ectopic membrane localization away from the apical domain (Figures 4H,J). Overexpression within the Rab11aDN background did not affect the localization of the Crb2aEXT protein as this transgene lacks the endocytic signal associated with the intracellular domain (Figure 4K). Conversely, in Rab11aDN retinas, the Crb2aFL localized to distinct puncta, consistent with aberrant recycling of the Crb2aFL protein back to the membrane (Figure 4I). As with Crb2a loss-of-function studies, we also assessed if the overexpression of Crb2a might affect the size of the apical domain in RPCs. Neither Crb2aFL nor Crb2aEXT resulted in a change to the apical domain area (Supplementary Figure 4K). Together, these data suggest that defective cell cycle exit observed in RPCs expressing either Crb2aFL or Rab11aDN may result from ectopic localization of the Crb2a intracellular domain to non-apical regions of the cell. Mechanistically, we suggest that the Crb2a intracellular domain may titrate binding partners away from the apical domain, thereby modulating multiple signaling pathways regulated by factors that bind the Crb2a intracellular domain (Figure 4A).

### Localization of Crb2a Intracellular Domain to Rab11a Recycling Endosomes Maintains RPCs in the Cell Cycle

**Figure 5:**
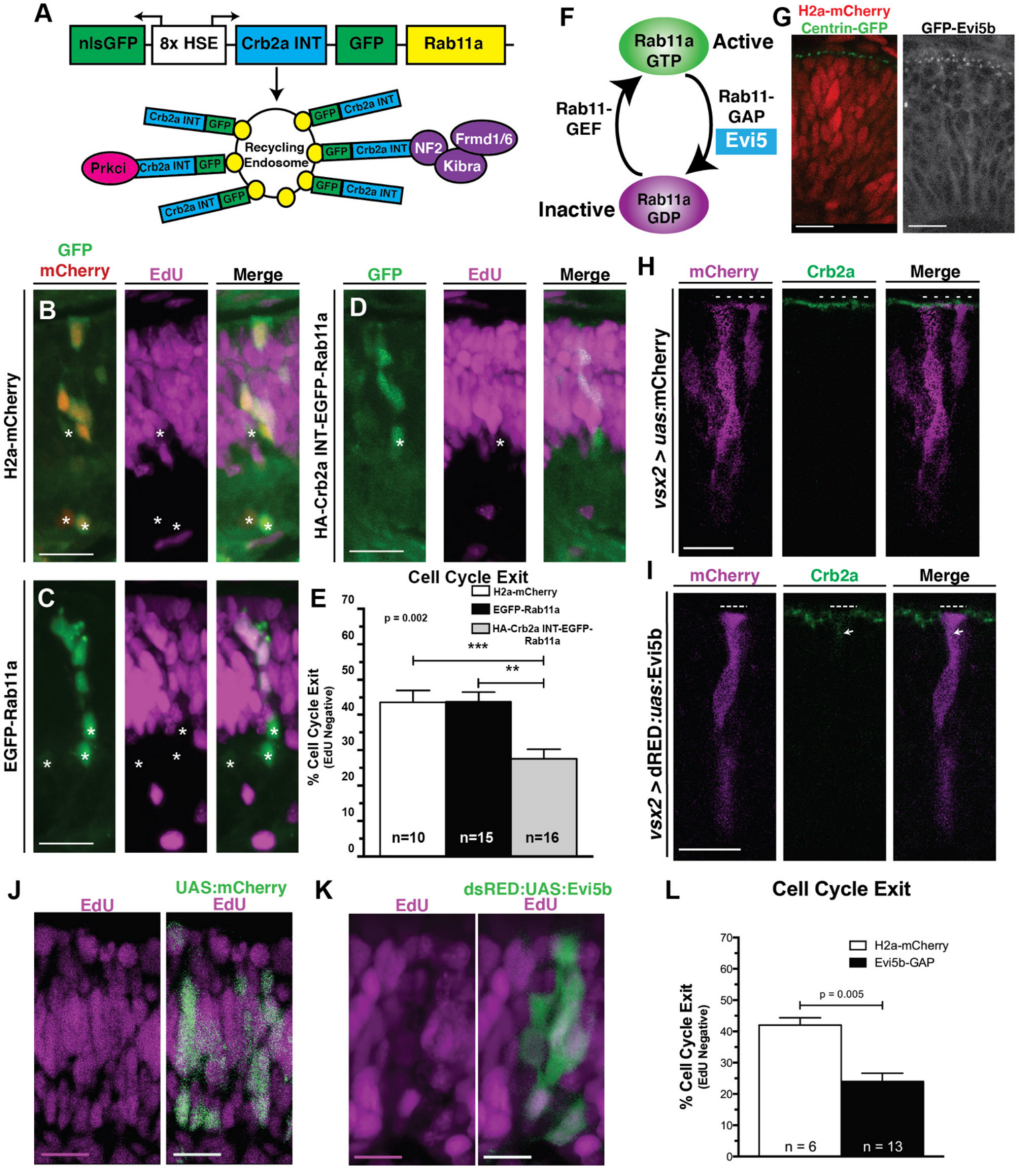
Crb2aINT localized to Rab11a recycling endosomes and inhibition of recycling endosome activity promote RPC proliferation. **(A)** Schematic of the heat-shock inducible transgene to localize the Crb2aINT to EGFP-Rab11a positive recycling endosomes. **(B–D)** Images assessing cell cycle exit (EdU negative) within clones of **(B)** Control (nlsGFP; H2a-mCherry). **(C)** EGFP-Rab11a overexpression (nlsGFP; EGFP-Rab11a) or **(D)** Crb2aINT localized to Rab11a-positive recycling endosomes (nlsGFP; Crb2aINT-GFP-Rab11a). Transgene expression is induced through heat-shock at 24 hpf, with EdU pulse from 36 to 48 hpf. **(E)** Quantification of average cell cycle exit across clones. Graph represents mean percentages of cell cycle exit across clones with error bars indicating SEM. Listed n-values represent number of quantified embryos within each genotype. Statistics are the result of a One-way ANOVA (*p* = 0.002) followed by a Tukey's multiple comparisons test. ***p < 0.001; **p < 0.01. **(F)** Schematic of the molecular switching of Rab11a from the GTP-bound active form to the GDP-bound inactive form. Hydrolysis of Rab11a-GTP to Rab11a-GDP is mediated by the Rab11a-GAP, Evi5. **(G)** Comparisons of centrosome localization (Centrin-GFP; left) to transgenic expression of a GFP-tagged Evi5b (right) in 28 hpf RPCs. Both transgenes localize in apical puncta, suggesting conserved localization of Evi5b to peri-centrosome regions within the developing zebrafish retina. **(H,I)** Representative (*n* > 5 embryos/genotype) immunostaining assessing Crb2a localization within 32 hpf RPCs after transgenic expression of either **(H)** Control (mCherry) or **(I)** Evi5b transgenes. Dotted lines in **(H,I)** indicate apical domains of transgenic cells, including regions where Crb2a staining is lost at the apical surface in Evi5b transgenic cells in I. Arrow in I indicates non-apical localization of Crb2a. **(J,K)** Assessment of cell-cycle exit through incorporation of EdU after a 12-h pulse from 36 to 48 hpf in **(J)** Control (UAS:mCherry) or **(K)** Evi5b transgenic clones, with transgene expression driven from by *vsx2*:Gal4 expression in RPCs. **(L)** Quantification of average cell-cycle exit (Edu-negative cells; 12 h pulse from 36 to 48 hpf) within transgenic clones of 48 hpf retinas. N's represent number of clones assayed from >4 animals/genotype. Bar graph in **(L)** represent the means with SEM, with statistics indicating the results of an unpaired Student's *t*-test. Scale bars represent 25 μm. * in **B–D** indicates cells counted as having exited the cell cycle (EdU negative).

To examine more directly the role of the Crb2a intracellular domain when internalized to Rab11a vesicles, we generated an additional Crb2a transgene. We fused the Crb2aIN to Rab11a itself (Crb2aIN-EGFP-Rab11a) (Figure 5A). Unlike Crb2aINT, the Crb2aIN-EGFP-Rab11a protein was stable and detected in RPCs. This scenario should mimic Crb2a association with recycling endosome compartments. Experimentally, we first investigated whether ectopically localized Crb2a intracellular domain could titrate binding partners away from the apical region of RPCs, as hypothesized (Supplementary Figure 5). We observed Prkci expression at punctate sites of EGFP-Rab11a recycling endosomes and HA immunoreactivity, suggesting a functional transgene (Supplementary Figure 5). Next, we assessed the effects of ectopically localized Crb2aIN on RPC proliferation. Significantly, the expression of the Crb2aIN-EGFP-Rab11a transgene resulted in decreased cell cycle exit compared with either H2a-mCherry or EGFP-Rab11a controls (Figures 5B–E), consistent with the intracellular domain of Crb2a being required for the proliferative phenotype observed with the overexpression of the full-length version (Figure 4). Importantly, fusion of the Crb2aIN did not alter EGFP-Rab11a localization or dynamics, as predominately apical EGFP-positive puncta were observed in RPCs (Supplementary Figure 5). Overall, these experiments suggest that mis-localized Crb2a in Rab11aDN retinas inhibits RPC differentiation, potentially through the modulation of signaling pathways associated with the Crb2a intracellular domain.

### The Rab11a-GAP, Evi5b, Is Apically Localized, Promotes Crb2a Mis-localization, and Inhibits RPC Cell Cycle Exit

To this point, we have inhibited recycling endosome activity through the overexpression of the Rab11aDN transgene. The changes in Crb2a localization and effects on neurogenesis in RPCs imply that the nuclear position-dependent concentration of recycling endosomes might activate endomembrane recycling within the apical region. How might this happen? Rab proteins are regulated in part by GAPs, which promote the GDP-bound inactive form of Rab proteins (Figure 5F). Several laboratories have characterized a Rab11-GAP, Evi5, that localizes to centrosome appendages (Dabbeekeh et al., 2007; Westlake et al., 2011; Hehnly et al., 2012; Laflamme et al., 2012), which in RPCs are anchored at the apical surface (Supplementary Figures 1E,F, Figure 5G). The centrosomal localization of Evi5 is intriguing, as this provides a potential mechanism whereby the concentration of Rab11a alters its activity through proximity to its apically localized GAP. To test the hypothesis that nuclear position-dependent concentration of endosomes corresponds to activity changes in Rab11a, which impacts both Crb2a localization and cell cycle exit, we assessed the consequences of manipulating Evi5. We first examined the localization of the zebrafish ortholog of Evi5 (Evi5b) within RPCs through the transgenic expression of a GFP-Evi5b fusion protein. Similar to previous reports of centrosomal appendage localization, we observed GFP-Evi5b localization in bright, punctated foci at the apical domain of RPCs (Figure 5G), reminiscent of centrosome localization as marked by Centrin-GFP (Figure 5G).

We next determined the consequence of altering Rab11a activity through Evi5b overexpression, focusing on protein trafficking and cell cycle exit. In control cells overexpressing mCherry alone, Crb2a immunoreactivity remained concentrated at the apical surface (Figure 5H). Consistent with our studies using the Rab11aDN transgene, the inhibition of Rab11a activity by Evi5b overexpression resulted in loss of Crb2a immunolocalization from the apical membrane and subsequent increase in internalized/mis-localized Crb2a expression (arrows in Figure 5I). The inhibition of Rab11a activity by Evi5b also resulted in reduced cell cycle exit as assessed by EdU incorporation from 36 to 48 hpf (Figures 5J–L). With the link between changes in Rab11a activity and Crb2a localization established as a mechanism influencing cell cycle exit of RPCs, we next evaluated whether signaling was altered, beginning with the Notch pathway.

### Rab11aDN Expression Results in an Autonomous Reduction in Notch Signaling Through Decreased Membrane Localization of the Notch Receptor

**Figure 6:**
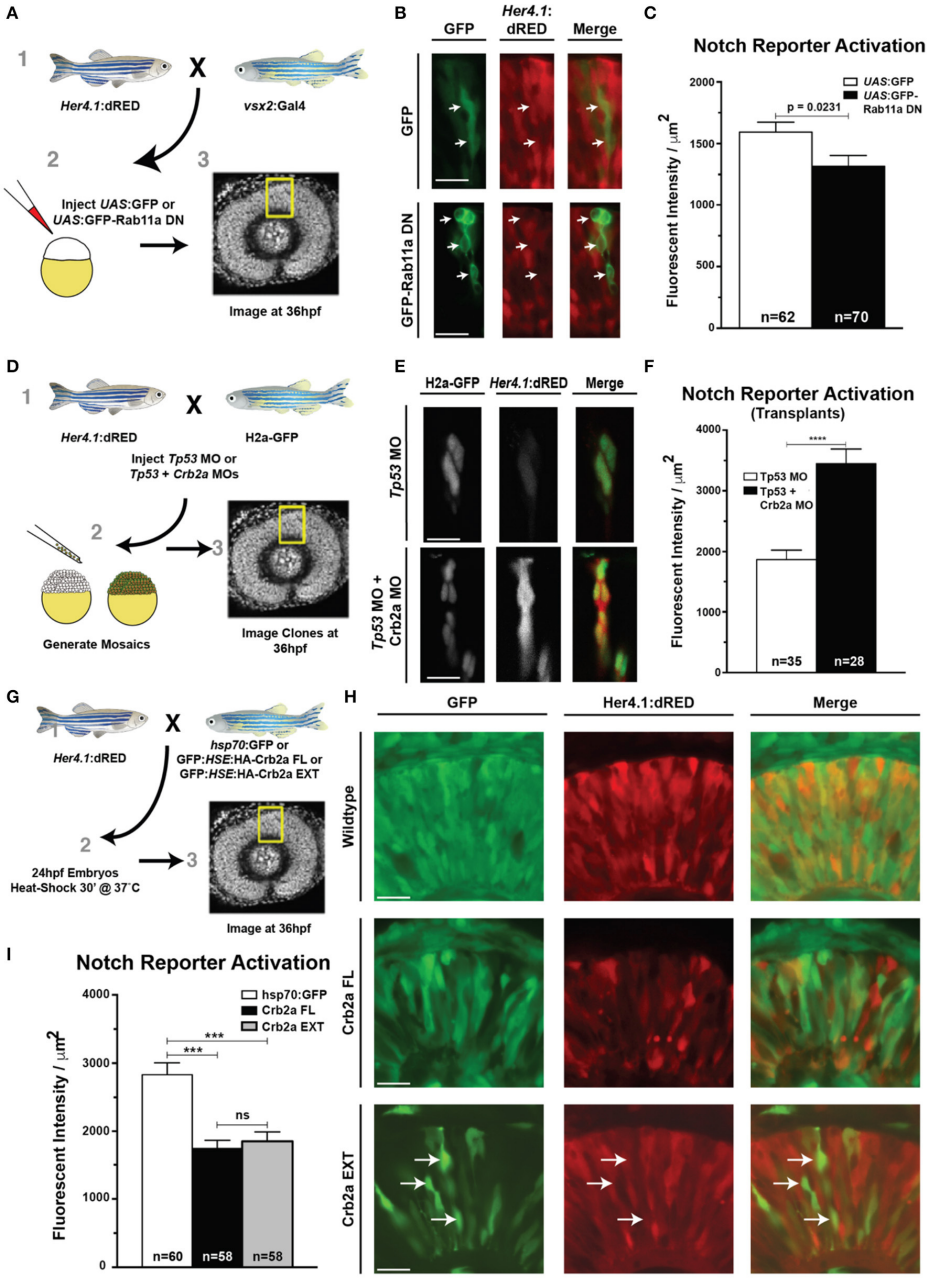
Rab11aDN expression and Crb2a function to inhibit Notch-reporter activation. **(A)** Schematic of experiments assessing the *her4.1*:dRED Notch reporter activity in control (UAS:GFP) or (UAS:GFP-Rab11aDN) injected embryos. **(B)** Representative images of Notch-reporter (*her4.1*:dRED) activity in control (top) and Rab11aDN (bottom) expressing cells. **(C)** Quantification of relative Notch-reporter (*her4.1*:dRED) fluorescence in GFP-labeled cells. **(D)** Schematic of genetic mosaic experiments for Control (*Tp53* MO), *her4.1*:dRED/H2a:GFP cells or Crb2a morphant (*Tp53* + *Crb2a* MO), *her4.1*:dRED/H2a:GFP to detect autonomous Notch-reporter activation within a wildtype background. **(E)** Example of Notch reporter (*her4.1:*dRED) activation in Control (top) and Crb2a morphant (bottom) clones. **(F)** Quantification of Notch reporter fluorescent intensities (*her4.1:*dRED) of Control and Crb2a morphant cells. **(G)** Schematic of experiments examining the consequence of heat-shock activation of control (GFP), Crb2a-FL, or Crb2a-EXT transgenes on Notch reporter (*her4.1:*dRED) activation **H)** Representative images of *her4.1*:dRED after expression of GFP, Crb2a-FL, Crb2a-EXT transgenes in 36 hpf retinas. Arrows in lower panels indicate locations of high Crb2a-EXT expressing cells that show low activation of the Notch reporter (*her4.1:*dRED) transgene. **(I)** Quantification of Notch-reporter activation in Control (GFP), Crb2a-FL, or Crb2a-EXT overexpressing cells. Bar graphs in **(C,F,I)** represent mean *her4.1*:dRED Notch reporter activation across individual cells, normalized per unit area, with error bars indicating SEM. Statistics in **(C,F)** are the result of an unpaired *t*-test, while statistics in **(I)** represent results of a One-way ANOVA followed by Tukey's Multiple Comparisons Test. ***p < 0.001; ****p < 0.0001. n-values in **(C,F,I)** represent number of cells counted from at least five different embryos for each experimental condition. Scale bars represent 25 μm.

The role of Notch signaling in regulating the balance between proliferation and cell cycle exit in retina neurogenesis is well-established (Moore and Alexandre, 2020). Furthermore, Notch activity correlates with nuclear position: within RPCs, Notch activity increases as the nucleus approaches the apical surface (Del Bene et al., 2008). We therefore probed whether changes to Rab11a or Crb2a altered Notch signaling through the use of Notch reporter transgenes. Using the Notch transgenic reporter line *her4.1*:dRED, which utilizes the regulatory sequence of the Notch target gene *her4.1* to express red fluorescent protein (Yeo et al., 2007), we mosaically expressed either GFP or GFP-Rab11DN in RPCs (Figure 6A). We measured a modest decrease in Notch activity in cells expressing the GFP-Rab11aDN (Figures 6B,C). The decrease in observed Notch reporter activation was curious for two reasons. First, reduction to Notch pathway activation in the neural retina is generally associated with elevated cell cycle exit (Riesenberg et al., 2009), yet the expression of Rab11aDN causes reduced cell cycle exit (Figure 3B). Second, loss of Crb2a, which occurs with the expression of Rab11aDN, is associated with increased Notch activity in zebrafish hindbrain neuroepithelia (Ohata et al., 2011). Potentially, however, Rab11aDN impacts Notch receptor trafficking, precluding its activation by secondary events, such as Crb2a internalization. Indeed, in *Drosophila* sensory organ precursor cells, Rab11 has been shown to mediate Notch trafficking (Emery et al., 2005; Huttner and Kosodo, 2005). To test if Rab11aDN expression affects the Notch receptor trafficking, we analyzed the localization of the Notch1a^Δ*E*^ transgene in Rab11aDN-positive cells. We previously reported that the sEGFP-Notch1a^Δ*E*^ transgene localizes to the membrane and accumulates in apical puncta within RPCs (Clark et al., 2012). *En face* imaging of the apical surface of RPCs confirmed the enrichment of puncta within the apical region (Supplementary Figures 6A,B). The expression of Rab11aDN within RPCs of Notch1a^Δ*E*^ transgenic embryos resulted in a loss of apical puncta (Supplementary Figures 6A–C). Rab11aDN expression had no effect on the membrane localization of a secreted EGFP with a GPI membrane anchor (Supplementary Figure 6D), suggesting that general membrane-associated protein trafficking was not affected in Rab11aDN RPCs. Together, these data suggest that low levels of Notch reporter activation in Rab11aDN RPCs result from altered Notch receptor trafficking.

Although the continual expression of Rab11aDN prevents Notch from reaching the plasma membrane, we suggest that endogenously, Rab11a activity is modulated in a nuclear-dependent fashion and therefore Notch would be trafficked to the apical plasma membrane and subsequently regulated by Crb2a internalization.

We therefore next assessed the significance of Crb2a expression on Notch activity within RPCs. To evaluate the consequence of Crb2a loss-of-function on Notch signaling independent of an overall tissue polarity defect caused by global loss of Crb2a in the retinal neuroepithelium (Malicki and Driever, 1999), we analyzed *her4.1*:dRED activation in genetic mosaics within control and Crb2a MO-injected embryos (Figures 6D–F). Consistent with previous reports in zebrafish hindbrain neuroepithelia (Ohata et al., 2011), Crb2a knockdown autonomously increased Notch reporter activation (Figures 6D–F). Additionally, the transgenic overexpression of Crb2aFL or the Crb2aEXT both resulted in an autonomous decrease in Notch pathway activation, confirming previous reports that the Crb2a extracellular domain is sufficient to inhibit Notch signaling in RPCs (Figures 6G–I; Ohata et al., 2011).

**Figure 7:**
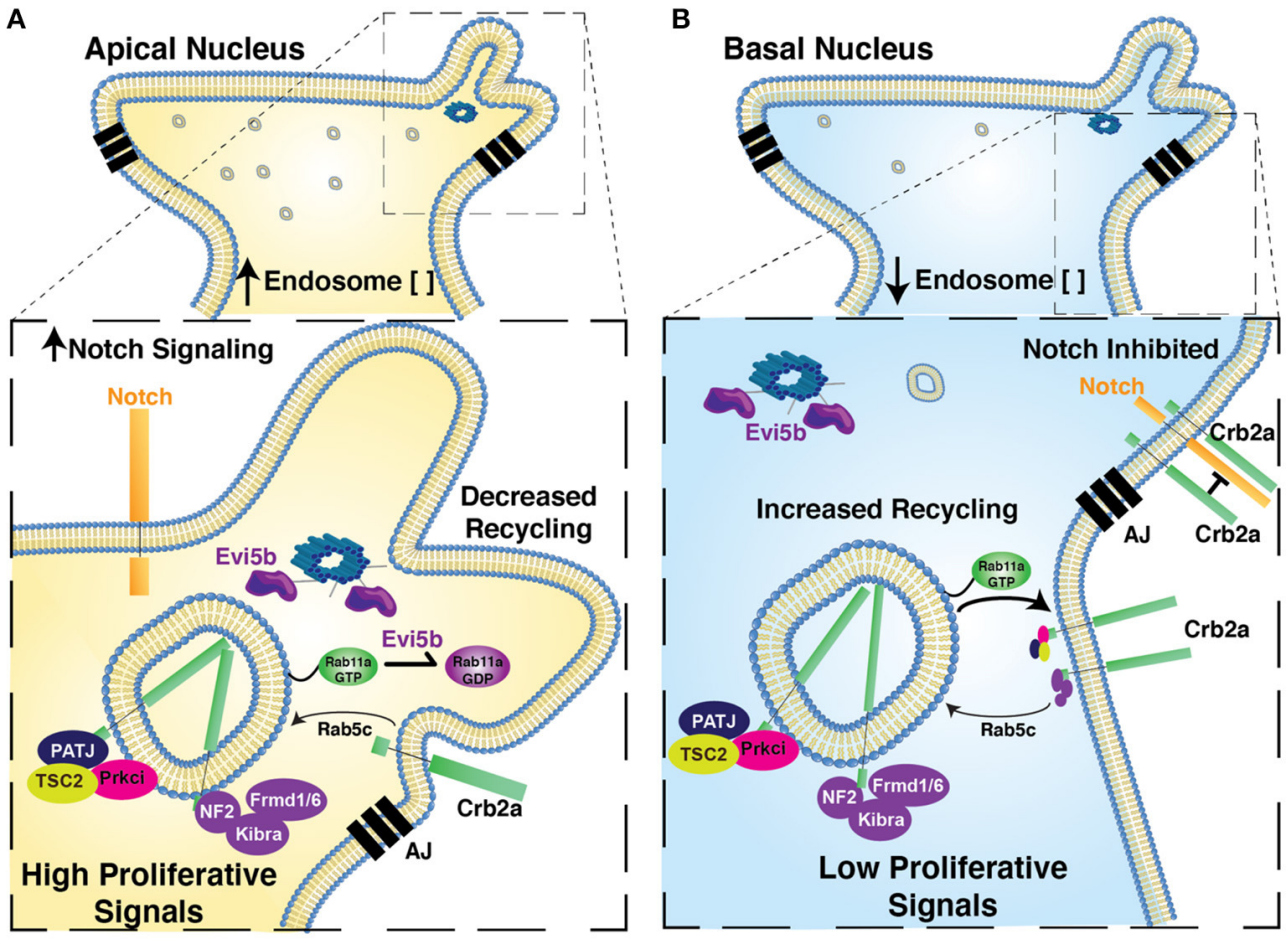
Proposed model for relationship of Nuclear Position, endosome activity, and the regulation of polarized signaling and retinal neurogenesis. Model figure suggesting the functional significance of nuclear position in the regulation of apical endosome concentration, Rab11a recycling endosome activity, the regulation of Crb2a apical protein localization and the regulation of autonomous signaling. In cells with apical nuclei **(A)** we suggest apical concentration of Rab11a-positive recycling endosomes biases a state in which Rab11a is preferentially in the inactive, GDP-bound form, due to the close proximity of apical recycling endosomes to the centrosome-localized Rab11a-GAP, Evi5b. This in turn decreases recycling of Crb2a to the apical junctions, thereby decreasing the *cis*-inhibition of Notch signaling by Crb2a (increased Notch signaling) and mis-localizing Crb2a interacting proteins from the apical junction. Combined these changes promote autonomous signaling to promote a proliferative state. In cells with basal nuclei **(B)** Rab11a is preferentially in a GTP-bound active state, promoting Crb2a recycling, inhibiting Notch-signaling, and maintaining apical localization of Crb2a-interacting proteins. In combination, this biases cells toward an autonomous signaling state that promotes neurogenesis. AJ, Apical Junctions.

Combined, our data are consistent with a causal relationship between nuclear migration and cell cycle exit (Baye and Link, 2007), mediated by nuclear position-dependent dynamic Rab11a activity that affects Crb2a internalization and thus cell signaling (Figure 7). Moreover, considering our observations of reductions in Notch reporter activation from either Crb2aFL or EXT transgenes (Figure 6), our data suggest that the modulation of Notch activity contributes to the relationship between nuclear migration dynamics and neurogenesis. However, we found that while Notch activity was affected by the extracellular portion of Crb2a (Figures 6H,I), the internal domain of Crb2a also had significant influence on retinogenesis (Figures 4G, 5E).

### Rab11aDN Expression Affects Multiple Signaling Pathways

**Figure 8:**
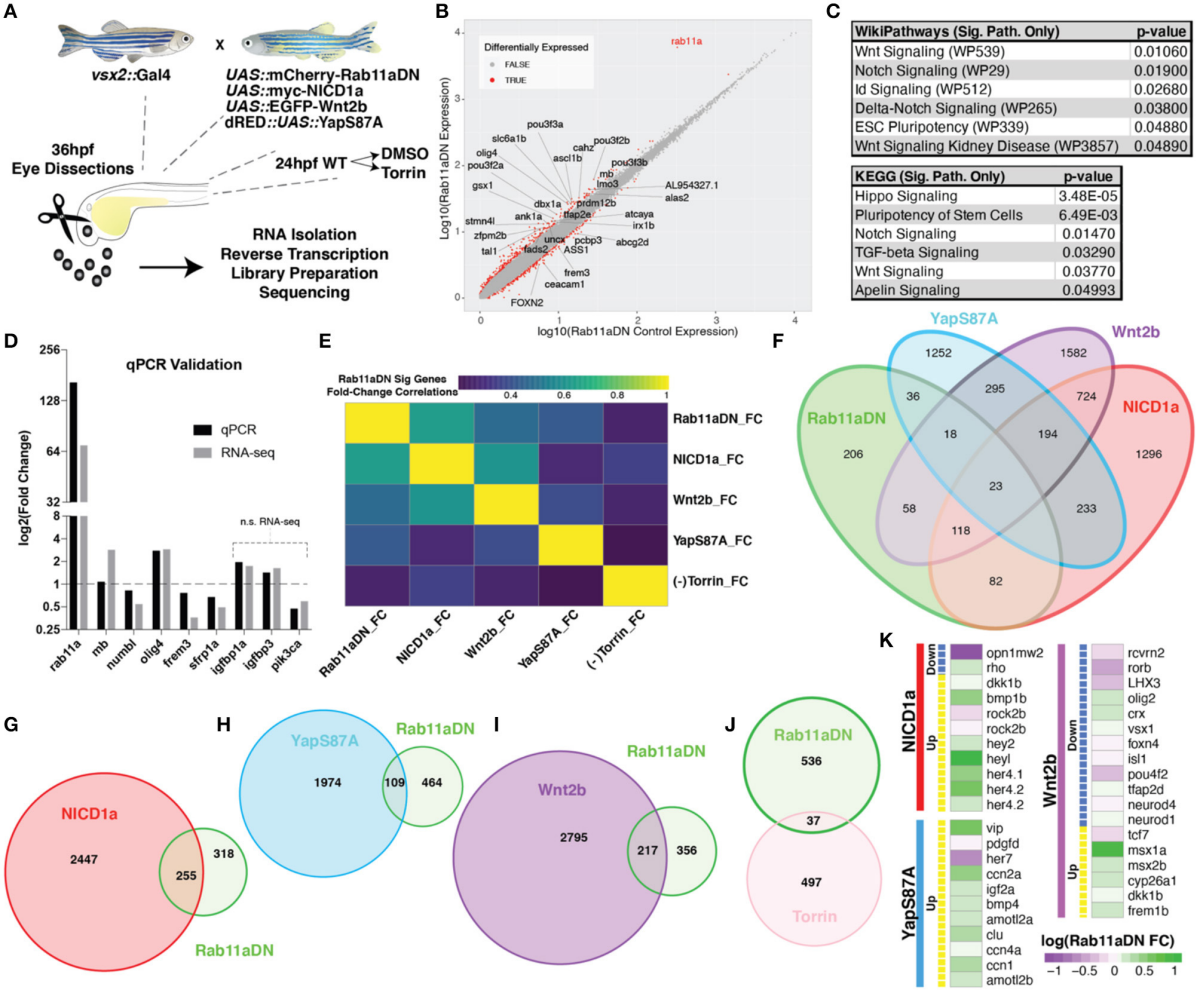
RNA-sequencing of Rab11aDN retinas identifies shared features with modulation of multiple signaling pathways control RPC proliferation. **(A)** Schematic of genetics for input for RNA-sequencing experiments to test changes in retinal transcript expression resulting from Rab11aDN expression, Notch-pathway activation (NICD1a), Wnt-pathway activation (Wnt2b), inactive Hippo pathway (YapS87A; Yap constitutive active), or inhibition of mTOR signaling (Torin). **(B)** Global analysis of differentially expressed transcripts in 36 hpf Rab11aDN retinas. Differentially expressed transcripts are indicated in Red. Gene names are listed for differentially expressed transcripts that display high residual to the mean. **(C)** Target pathways from IPA pathway analysis on Rab11aDN differentially expressed transcripts using WikiPathways (top) and KEGG pathways (bottom). **(D)** qRT-PCR validation of RNA-sequencing results for transcripts associated with the Notch (mb and numb), Hippo (olig4 and frem3), Wnt (sfrp1a), and mTOR (igfbp1a, igfbp3, pik3ca) pathway modulations. **(E)** Correlation of the fold changes of differentially expressed transcripts from Rab11aDN experiments with the fold changes observed in additional RNA-seq samples, indicating similarity between transcript signatures. **(F–J)** Venn diagrams of differentially expressed transcripts across RNA-sequencing samples of **(F)** All RNA-sequencing samples performed or pairwise comparisons of Rab11aDN differentially expressed transcripts with **(G)** NICD1a, **(H)** YapS87A, **(I)** Wnt2b overexpression studies, or **(J)** inhibition of mTOR signaling using the Torin inhibitor. **(K)** Heatmaps of Up- (Yellow) or Down-regulated transcripts within NICD1a, YapS87A, or Wnt2b experiments indicating the corresponding expression fold change in Rab11aDN studies.

To evaluate whether pathways in addition to Notch are affected by altered Rab11a activity, we performed RNA-sequencing analysis on dissected eyes from 36 hpf control and Rab11aDN embryos (Figure 8A). The transcript displaying the highest fold change in Rab11aDN embryos was Rab11a, consistent with our transgenic overexpression of Rab11aDN (Figure 8B, Supplementary Table 1). Differential expression analysis indicated that 573 (adjusted *p* < 0.05) transcripts showed altered expression between control and Rab11aDN embryos (Figure 8B, Supplementary Table 1). Ingenuity pathway analysis (IPA; Ingenuity Systems, Redwood City, CA, USA) of Rab11aDN differentially expressed transcripts using both WikiPathways and Kegg pathways comparisons suggested that changes in Rab11a activity affect numerous signaling pathways including Notch (as expected), but also Wnt, Id, Hippo, Tgf-beta, and Apelin-mTOR activities (Figure 8C, Supplementary Figure 7A). These broad, literature-based analyses led us to further explore these pathways in depth. We first used quantitative reverse transcription (RT)-PCR to validate transcript changes associated with the various signaling networks highlighted in the pathway analyses (Figure 8D). Next, to assess relationships of Rab11aDN transcriptional signatures to signaling pathways that are activated/inhibited specifically within the developing zebrafish retina, we compared Rab11aDN RNA-sequencing experiments to scenarios in which we acutely modulated Wnt, Notch, Hippo, or mTOR signaling using *vsx2:*Gal4;*UAS:*EGFP-Wnt2ba, *vsx2:*Gal4;*UAS*:myc-NICD1a (Scheer et al., 2001), and *vsx2:*Gal4;dsRED*:*UAS*:*YapS87A (Miesfeld and Link, 2014) transgenic animals or through the addition of the mTOR inhibitor, Torin, respectively (Supplementary Table 1, Supplementary Figure 8). We performed similar RNA-sequencing experiments on 36 hpf dissected retinas from larvae of control, Wnt2b overexpression, NICD1a overexpression experiments, or treatment of embryos with 100 μM Torin. Data assessing changes in Hippo signaling were obtained from our published studies assessing the consequence of YapS87A overexpression in 36 hpf retinas within the same experimental setup (Miesfeld et al., 2015). To examine the extent to which Rab11aDN differentially expressed transcripts displayed similar alterations in expression when these signaling pathways were activated/repressed, we assessed both correlations of fold changes of Rab11aDN differentially expressed transcripts across samples (Figure 8E) and the degree to which individual transcripts were differentially expressed across the multiple experimental paradigms (Figures 8F–K). In general, transcripts that were differentially expressed in Rab11aDN experiments displayed the most congruent expression changes to experiments where the Notch signaling pathway was activated (Figures 8E–G, Supplementary Figures 7B–E). Specifically, numerous Notch pathway targets (*hey2, heyl, her4.1, her4.2*) were up-regulated within Rab11aDN experiments (Figure 8K). However, many of the differentially expressed transcripts in YapS87A or Wnt2b overexpression experiments also showed consistent changes within Rab11aDN retinas (Figure 8K).

Our data, altogether, suggest an active role of Rab11a recycling endosomes in the regulation of multiple signaling pathways. As studies of Crb1/2 knockout mouse retinas lead to both a proliferative phenotype and alterations to multiple signaling pathways including the Notch, Hippo, and p120-catenin pathways (Alves et al., 2013; Pellissier et al., 2013), we conclude that Rab11a activity is impacted by nuclear position in RPCs, and thus, affects Crb2a localization, which leads to the modulation of several signaling pathways that together influence retinal neurogenesis.

## Discussion

In this study, we explored the potential of polarized endocytosis to regulate the relationships between nuclear position, cell signaling, and neurogenesis within the zebrafish retina to gain insight into how cellular features, such as nuclear position, can translate to a transcriptional signature that drives cell cycle exit. In RPCs, we found that early and recycling endosomes are concentrated in a nuclear position-dependent manner, situating localized endomembrane activity as a potential regulator of cell signaling that influences neurogenesis. We tested the effect of Rab11aDN expression to address whether recycling endosome function in particular is required to mediate these relationships. The expression of the Rab11aDN transgene caused a redistribution of the Crb2a protein from cell junctions and the apical plasma membrane to intracellular puncta, with overall cell polarity maintained. The analysis of cell cycle exit of RPCs in Rab11aDN retinas revealed an increased proportion of RPCs remaining in the cell cycle, which was phenocopied by Crb2a loss-of-function or with Crb2a mis-localization. Previous studies have shown a requirement of the Rab11-interacting proteins (Rab11-FIP) in the regulation of the inner nuclear layer (INL) cell differentiation in both mouse and zebrafish (Muto et al., 2006, 2007), and of relevance to our studies, knockdown of Rab11-FIP4 results in smaller eyes due to aberrant cell proliferation and cell cycle exit. Here, we provide evidence for a direct role of Rab11a in retinal neurogenesis. Importantly, we provide additional support for nuclear position-dependent localized activity of endosome recycling through the examination of the Rab11a-GAP, Evi5b. Consistent with previous reports, the GFP-Evi5b transgene localized at puncta near the apical surface, where centrosomes are anchored. Evi5b overexpression also resulted in redistribution of Crb2a protein and reduced cell cycle exit, phenotypes akin to those observed with Rab11aDN expression. These results support a hypothesis that recycling endosome activity is inhibited by promoting the Rab11a-GDP-bound state when recycling endosomes are apically positioned and in close proximity to the centrosome. As we observed an apical concentration of recycling endosomes in RPCs with apical nuclei, we suggest that Rab11a and recycling endosome activity is regulated in a nuclear position-dependent manner (Figure 7). The role of localized endocytosis and recycling of transmembrane and junction associated proteins has been demonstrated previously. For example, G protein-coupled receptor signaling (Weinberg and Puthenveedu, 2019) and several pathways controlled by AMOT, a cell junction associated signaling factor (Heller et al., 2010; Cox et al., 2015; Brunner et al., 2020), are regulated through locally concentrated endocytic activity. However, the activity of an endocytic cycle has not been shown previously to be associated with nuclear position.

Given the novelty of this finding, we further explored the mechanism by which Rab11aDN inhibits RPC differentiation through examining the consequence of Rab11aDN expression on RPC signaling pathways. Rab11aDN expression caused impaired localization of a Notch transgenic protein and concomitant reduction in Notch reporter activation. However, the modulation of Crb2a expression levels alone through gain or loss-of-function experiments suggests that Crb2a inhibits Notch signaling autonomously, consistent with previous reports (Ohata et al., 2011). Therefore, we suggest that Rab11aDN expression changes the localization of the Crb2a protein, consistent with the intracellular accumulation of Crb2a immunofluorescence and redistribution of Crb2a binding partners that regulate numerous signaling pathways. Additionally, the localization of the Crb2a intracellular domain to EGFP-Rab11a vesicles caused an increase in RPC proliferation, a phenotype that should be independent of the direct *cis* inhibition of the Crb2aEXT on the Notch receptor. This supports a mechanism by which redistribution of Crb2a modulates signaling for numerous pathways. Our transcriptomic analyses are in agreement with this notion: Rab11aDN significantly shifted signatures of several pathways known to influence retinal neurogenesis including Notch, Wnt, and Hippo, and to a lesser extent mTOR signaling.

One remaining question is what complements Rab11 activity to mediate Crb2a recycling between the apical cell surface to internalized endosome vesicles? A potential clue comes from our studies investigating Rab5 (Supplementary Figure 3E). The expression of Rab5 constitutive-active protein results in Crb2a accumulation in large vesicles, confirming a significant role for endosomal trafficking of Crb2a (Lu and Bilder, 2005; Roeth et al., 2009; Clark et al., 2011). Furthermore, Crumbs proteins interact with components of the evolutionarily conserved retromer complex that facilitates protein transport back to the *trans*-Golgi network (TGN) (Pocha et al., 2011; Zhou et al., 2011). Many retromer-associated proteins will accumulate at the TGN; however, this is not the case for *Drosophila* Crumbs, suggesting an active recycling mechanism back to the apical domain (Pocha et al., 2011). While not yet experimentally analyzed, it was postulated that the passage of Crumbs through the TGN may facilitate co-transport of an apically secreted, Crumbs binding partner (Pocha and Knust, 2013). A similar mechanism is observed for Wg secretion by Wntless in *Drosophila*, where Wntless binds Wg in the TGN to facilitate secretion, and Wntless is then re-internalized from the plasma membrane through endocytosis and trafficked by the retromer back to the TGN to renew the process (Franch-Marro et al., 2008; Port et al., 2008). From our data, we suggest minimally that Rab11a activity is important for apical recycling of Crb2a in RPCs. The details of other modulators of Crb2a will be important to assess as future studies.

Crb2a may not be the only apical junction associated protein whose localization is affected by endocytic recycling and which can influence neurogenic signaling. Two genes that displayed differential transcript abundance in Rab11aDN vs. control retinas provide possible insight to factors affected by Rab11a-mediated endocytosis and signaling: Vangl2 and Amotl2a. Work examining planar cell polarity, controlled by non-canonical Wnt signaling, has shown that Vangl2 targets Rab11(+) recycling endosomes to the apical domain to localize PCP determinants (Mahaffey et al., 2013). Research using zebrafish has shown that Amotl2 negatively regulates Wnt signaling by trapping β-catenin in Rab11 endosomes, thus reducing both cytoplasmic and nuclear accumulations of β-catenin (Li et al., 2012). Combined, these experiments suggest a requirement of Rab11 recycling endosomes for proper control of Wnt signaling. Amotl2 can also impact Lats kinase activity (Mana-Capelli and McCollum, 2018) and is itself a target of the transcriptional co-activator Yap (Calvo et al., 2013), providing a possible explanation for altered Hippo signaling as well.

Interestingly, the relationships between fundamental cellular processes and neurogenesis vary across species and in different parts of the nervous system (Willardsen and Link, 2011). For example, the length of the cell cycle has been shown to regulate mouse cortical neurogenesis (Lange et al., 2009; Pilaz et al., 2009). However, within the retina, this relationship does not exist (Baye and Link, 2007; Gomes et al., 2011). Inheritance of the mother centrosome is another fundamental cellular event that can influence neurogenesis. In *Drosophila* neuroblasts, inheritance of the mother centrosome at cell division cues cell cycle exit, whereas cell receiving the daughter centrosome remains proliferative (Conduit and Raff, 2010; Januschke and Gonzalez, 2010). This basic mechanism is conserved in mouse cortical progenitors, although the relationship between mother–daughter centrosome inheritance and neurogenesis is switched, and the bias on cell cycle exit or proliferation is less dramatic (Wang et al., 2009). It will be interesting to see whether the influence of polarized endocytic activity on neurogenesis described here is a conserved feature across tissues and species.

In summary, we suggest that nuclear position-dependent polarization of Rab11a can regulate the signaling networks to mediate the relationship of nuclear position and neurogenesis, at least partially through the regulation of Crb2a localization. While several individual pathways that regulate the proliferative capacity of RPCs are mis-regulated in Rab11aDN retinas, it is unlikely that one pathway is solely driving the proliferative phenotype. Instead, we propose that the combined transcriptional landscape in Rab11aDN retinas is collectively biasing RPCs to remain proliferative.

## Materials and Methods

### Zebrafish Transgenic Lines

Transgenic lines used in this study are listed in Supplementary Table 3. Tol2-Gateway constructs used throughout the course of these studies are listed in Supplementary Table 4.

Transgenic constructs were generated through Gateway® (Invitrogen, Carlsbad, CA, USA) recombination techniques into the Gateway-Tol2 as previously described (Kawakami, 2005; Kwan et al., 2007). The Crb2a entry clones were received from Abbie Jensen (UMASS-Amherst). The human Fzd5 enhancer (Willardsen et al., 2009) from the pG1-cfos-hFzd5CSA:GFP construct (gift from M. Vetter, University of Utah) was used to generate a 5′ entry clone by digesting with SalI and BamHI to remove the hFzd5CSA enhancer. The Fzd5 enhancer fragment was then ligated into the p5E-MCS gateway construct. The Tg(*tr*β*2*:EGFP)^*mw*59^ line was generated using the −1.8 kb *tr*β*2* promoter driving EGFP, followed by 2.0 kb of the *tr*β*2* Intron1 as described in Suzuki S. C. et al. (2013). Additional constructs used throughout the study are listed in Supplementary Table 3.

### Morpholinos

The following morpholino oligonucleotides were synthesized by GeneTools (Philomath, OR, USA): *tp53* MO, 5′-GCGCCATTGCTTTGCAAGAATTG-3′ (Robu et al., 2007) and MO2-*crb2a*, 5′-ACGTTGCCAGTACCTGTGTATCCTG-3′ (Omori and Malicki, 2006). Morpholinos were injected into 1–2 cell stage embryos. The efficacy of the splice-blocking morpholino (MO2-*crb2a*; Figure 2H) was determined using RT-PCR on control and injected embryos, assaying for inclusion of *crb2a* Intron 5 in RNA transcripts as previously described (Omori and Malicki, 2006).

### Antibodies

The following antibodies were used: phospho(ser10)histone3 [rabbit polyclonal, 1:1,000, Upstate Biologicals (Lake Placid, NY, USA), Cat#06-570], b-catenin [mouse monoclonal, 1:500, BD Biosciences (San Jose, CA, USA), Cat#610153], aPKC-i/z C20 (Prkci) [rabbit polyclonal, 1:1,000, Santa Cruz Biotechnology (Santa Cruz, CA, USA), Cat#SC-216)], Crb2a/Zs4 antigen [1:20, University of Oregon Monoclonal Antibody Facility (Hsu and Jensen, 2010)], Laminin [rabbit polyclonal 1:500, Sigma, Cat#L9393], Zrf-1 (GFAP) [mouse monoclonal, 1:5,000, Zebrafish International Resource Center (ZIRC)], Zpr1 (Arr3a) [mouse monoclonal, 1:250, Zebrafish International Resource Center (ZIRC)], and Zpr3 (Fret11) [mouse monoclonal, 1:250, Zebrafish International Resource Center (ZIRC)]. Immunofluorescence was performed on 4% paraformaldehyde fixed, whole embryos at indicated timepoints as previously described (Clark et al., 2011).

### Histology and TEM

Retinal histology and TEM were performed as previously described (Soules and Link, 2005).

### EdU Analysis

For EdU experiments, embryos were injected with 2 mM EdU into the pericardial region of both the experimental and sibling control embryos between 34 and 36 hpf. Embryos were grown to 48 hpf (12-h EdU pulse), then fixed in 4% paraformaldehyde overnight at 4°C, and processed for cryo-sectioning. Then, 10–12 μm sections were obtained on Superfrost® Plus (Fisher Scientific, Waltham, MA, USA) slides, and sections were allowed to dry for 1–2 h on the slides at room temperature (RT) prior to EdU detection. EdU incorporation was detected per manufacturer's instructions using 250 μl Click-iT reaction cocktail/slide. Nuclei were counter-stained using ToPro®-III iodide (642/661) (Molecular Probes, cat. #T3605) (1:10,000) in phosphate buffered saline (PBS).

### Blastula Transplantation

Chimeric embryos were generated through blastula transplantation as previously described (Carmany-Rampey and Moens, 2006).

### Quantification of Organelle Localization

Blastula transplants of EGFP-Rab (endosome marker) or Golgi (Man2a-GFP) and H2a-mCherry (nuclei) were performed to isolate small clones of labeled cells within the developing retinas. Confocal images of 28 hpf embryos were performed to assess nuclear position and endosome localization. Cells were binned by distance of the nuclei from the apical surface as a percent of total apical–basal distance. Endosome positioning was performed in a similar manner. Data represent quantification of endosomes from >10 cells/bin (apical, medial, and basal nuclei), with >5 embryos/genotype.

The analysis of centrosomes (Centrin-GFP) was performed in a similar manner, expect that only eight individual cells from >3 embryos were quantified as centrosome positioning within 28 hpf RPCs was always observed at the apical surface.

Transplants using the mitochondrial reporter (CoxVIII-GFP) were performed and imaged in a similar manner on six cells from >3 embryos. Quantification of mitochondrial positioning was assessed through quantification of fluorescent intensity using a line-scan across the entire apical–basal length of clonal RPCs (Supplementary Figures 1H,I). As we observed relatively uniform positioning of the mitochondrial network across the apical basal access, we compared the distribution of mitochondrial fluorescence both apical and basal to the center point of the nucleus and compared these proportions to a hypothetical uniform distribution using a linear regression to determine if the slopes and intercepts of the trendlines of mitochondrial fluorescence were significantly different from the hypothetical uniform distribution.

### Measurements of Apical Junction Length

Length of the electron dense junctions within TEM images was performed using blinded images. In cases where two junctions were present for individual RPCs, junctional length for the cell was averaged across the two junctions. In cases where a single junction was observed, the single junction length was used. Junctional length was assessed for 12 cells from >3 embryos for each genotype.

### Quantification of Notch Reporter Activity

Notch reporter transgene expression was determined through calculations of the average fluorescent pixel intensity of either the *her4.1*:dRED or the *tp1*:d2GFP reporters. Fluorescent intensity was averaged for unit area, with measurements taken within the regions defined by the nucleus.

### Heat-Shock Expression of Transgenes

Heat-inducible transgene expression was obtained through 30-min incubations of embryos in fish water at 37°C. Transgene expression was observed within 2–4 h, with phenotypic analyses conducted >8 h post-heat-shock induction (32–36 hpf).

### Cell Death Analysis

Embryos were incubated in 5 μg/ml acridine orange (Sigma-Aldrich) for 20 min at 28.5°C at 24, 48, or 72 hpf. Embryos were washed three times in fish water, anesthetized in tricaine, and embedded in 1% low-melt agarose in a glass-bottomed Petri dish for confocal imaging.

### Determination of Apical Domain Size

Apical domains of RPCs were obtained using a dorsal mount for imaging of 24–28 hpf embryos. Confocal imaging through the brain and retinal pigment epithelium (RPE) is performed to determine the positioning of confocal z-planes relative to the apical surface. The surface area of RPCs not undergoing mitosis (very large cells with more rounded shapes; Clark et al., 2012) is determined by outlining cells of interest to quantify the apical area, as performed previously in Clark et al. (2012).

### Determination of Prkci Fluorescence Recovery

Blastula transplants of control (Crb2a morpholino injected; hsp70:Gal4/UAS:GFP) or Crb2a heat-shock inducible transgenes (Crb2a morpholino injected; GFP:HSE:HA-Crb2aFL/EXT) were performed into wild-type hosts. Heat-shock induction of transgenes was performed at 24 hpf with embryos fixed at 36 hpf and processed for immunofluorescence. Line scans across the apical surface were used to determine “average Prkci fluorescence” of host tissue cells neighboring integrated clones. Percent recovery was determined based on the comparison of average Prkci fluorescent intensity of integrated clonal cells from control or Crb2aFL/EXT donors in GFP-positive regions across the line scan at the apical surface to average Prkci intensity of neighboring cells. Quantification was performed on >10 clones from >5 embryos for each genotype.

### Torin Treatment

Torin-1 was diluted in dimethylsulfoxide (DMSO) and added to the water of 24 hpf wild-type larvae at a final concentration of 100 μM. Control embryos were obtained by adding the equivalent volume of DMSO without Torin-1 to the fish water.

### Retinal RNA Extraction and Purification

Whole eyes were dissected from 36 hpf experimental and sibling control breedings from Torin treatment experiments or from *vsx2*:Gal4-driven transgenic expression of either *UAS*:mCherry-Rab11aDN, *UAS*:EGFP-Wnt2ba, and *UAS*:myc-NICD1a. Dissected retinas were immediately frozen on dry ice until ~60 pooled retinas per replicate were obtained for each genotype. RNA samples were collected in triplicate for each genotype. RNA was purified as described in Uribe et al. (2012) except that RNA was eluted in a 50 μl final volume. RNA quality was determined using an Agilent BioAnalyzer, and only samples displaying RNA integrity scores >7.5 were being used for library preparation and sequencing. Data for 36 hpf *vsx2*:Gal4>dsRED:*UAS*:YapS87A experiments generated in a similar manner were obtained from GSE71681 (Miesfeld et al., 2015).

### RNA-seq

A 50-bp single read sequencing was performed in triplicate for each genotype using an Illumina HiSeq2000 at VANTAGE (Vanderbilt University, Nashville, TN, USA) and is available using GEO accession GSE154895. RNA-sequencing files were aligned to the zv11 reference genome using STAR v2.7.1a (Dobin et al., 2013). YapS87A RNA-sequencing results (Miesfeld et al., 2015) were obtained from GSE71681 and re-aligned to zv11 for consistency across samples. Aligned reads were cleaned and sorted using samtools v1.9. Aligned reads were then assigned to genes and quantified using htseq v0.12.4 (Anders et al., 2015). Data normalization and differential expression analysis were performed using edgeR (Price et al., 2019).

## Data Availability Statement

Raw and processed RNA sequencing datasets are available within the GEO online repository under accession numbers GSE71681 and GSE154895. Additional inquiries on data availability should be directed to the corresponding author.

## Ethics Statement

The animal study was reviewed and approved by Medical College of Wisconsin, IACUC ID AUA1378. Written informed consent was obtained from the owners for the participation of their animals in this study.

## Author Contributions

BC and BL designed all the experiments, prepared the manuscript, and collected and analyzed the data. JM, MF, and RC collected and analyzed the data. All authors contributed to the manuscript editing and approved the final version.

## Conflict of Interest

The authors declare that the research was conducted in the absence of any commercial or financial relationships that could be construed as a potential conflict of interest.
