# Supplementary material for: Dynamic Polarization of Rab11a Modulates Crb2a Localization and Impacts Signaling to Regulate Retinal Neurogenesis
Source: Front Cell Dev Biol. 2021 Feb 9;8:608112. doi: 10.3389/fcell.2020.608112 (PMC7900515; doi:10.3389/fcell.2020.608112)
Supplement: Supplementary file 1 [file Data_Sheet_2.DOCX]

**Supplemental Figure 1.**


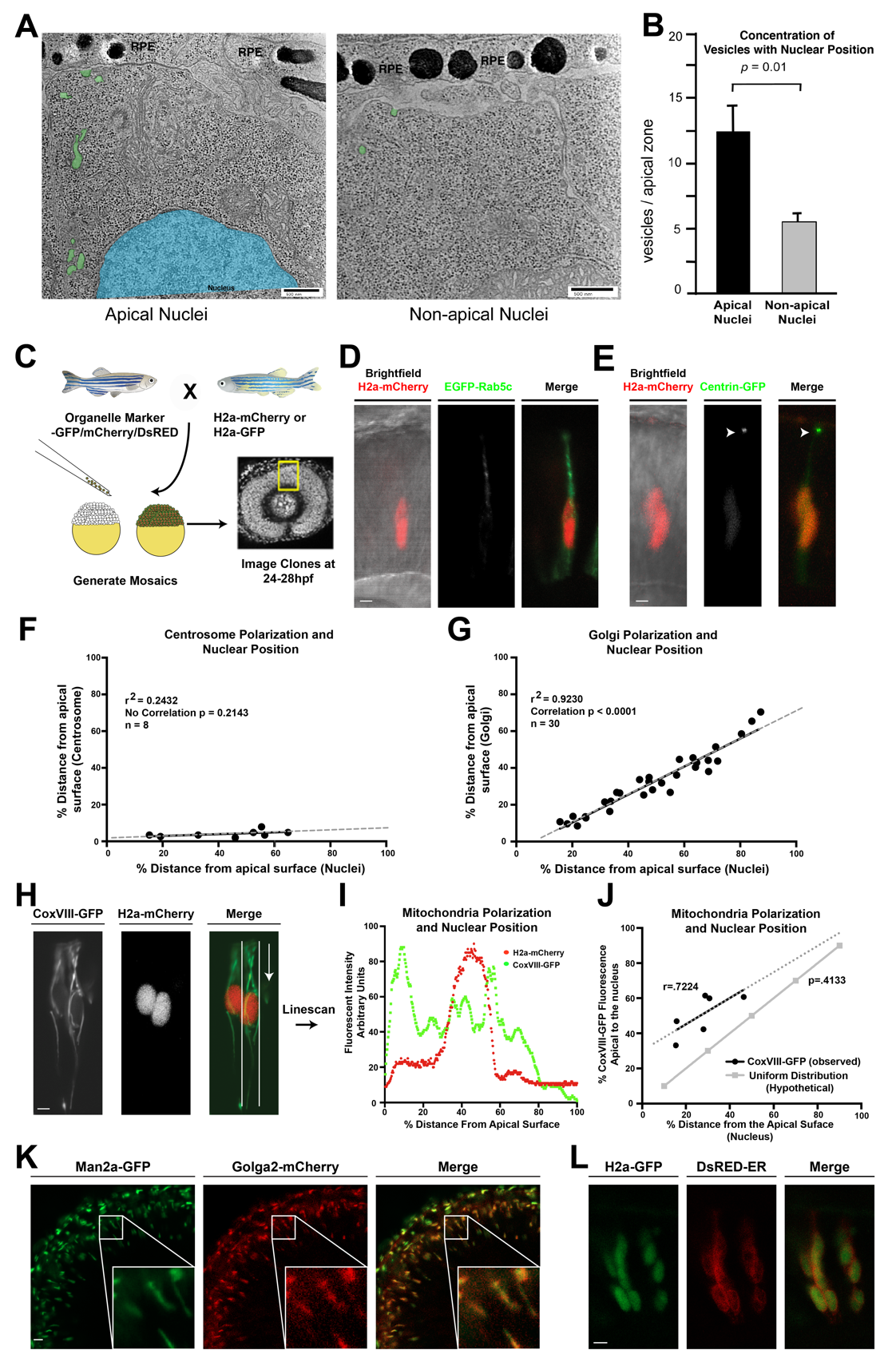


**Supplemental Figure 1. Quantification of organelle position during interkinetic nuclear migrations. A)** TEM of 34hpf RPCs with an apical nucleus (left) or basal nucleus (right). RPE – Retinal Pigemented Epithelium. Endosomes are pseudo-colored in green and nuclei in blue **B)** Quantification of the number of vesicles within apical regions of TEM images in RPCs with either apical or non-apical nuclei. Data represent the average number of vesicles observed in images across >5 cells/category. Scale-bars in TEM images represent 500nm. **C)** Schematic of genetic mosaic experiments used to determine organelle positioning with respect to nuclear position **D-E)** Representative images of **D)** early endosome (EGFP-Rab5c) localization in cells with basal nuclei or **E)** centrosome positioning (Centrin-GFP) with respect to nuclear position (H2a-mCherry). **F-G)** Graphs representing the relationship of **F)** centrosome (Centrin-GFP) or **G)** golgi (Man2a-GFP) and localization with respect to nuclear positioning. Statistics are results of a Pearson’s correlation with trendlines plotted from linear regressions of the data. Centrin-GFP labeled centrosomes (F) remain at apical surface regardless of nuclear position. Man2a-GFP labeled Golgi display a tight correlation with nuclear position (G), with localization apical to the nucleus. **H)** Representative images of genetic mosaics to assess mitochondrial (CoxVIII-GFP) localization with respect to nuclear position. **I)** Sample line-scan trace of nuclear (Red) and mitochondrial protein fluorescence within an individual RPC. Percentages of fluorescence apical to the nucleus were determined by recording the amount of fluorescence detected apical to the maximal peak fluorescence intensity of the nucleus. **J)** Quantification of mitochondrial polarization with respect to nuclear position (black circles and trendline) compared to a uniform distribution (grey squares and line) across the apical-basal axis. Results suggest no significant difference of mitochondrial reporter (CoxVIII-GFP) localization within RPCs compared to a hypothetical uniform distribution. **K)** Co-localization of markers for the medial (Man2a-GFP) and *cis*- (Golga2-mCherry) Golgi. **H)** Examples of genetic mosaics in which nuclei are labeled with H2a-GFP and the endoplasmic reticulum (ER) is labeled using ER targeting and retention sequences added to DsRED (DsRED-ER). The DsRED-ER reporter of ER localization was always observed to be perinuclear, regardless of nuclear positioning. Scale bars represent 10µm.

**Supplemental Figure 2.**

**
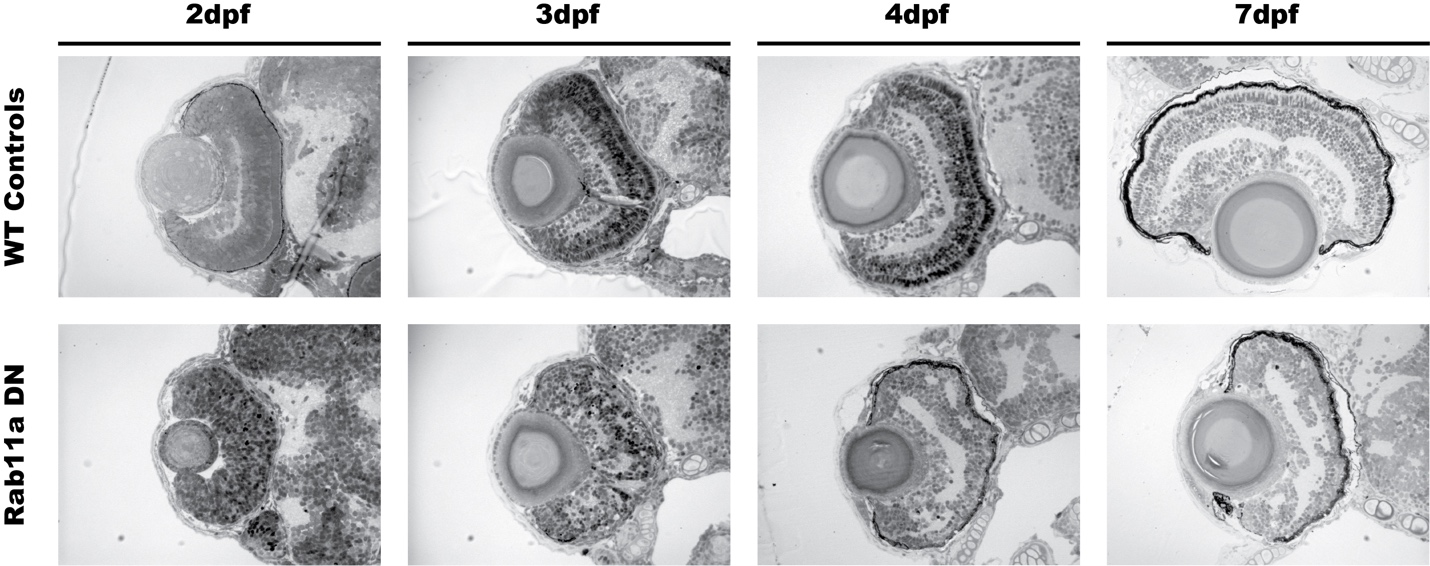
**

**Supplemental Figure 2. Rab11aDN expression alters retinal organization across development.** Retinal histology of sibling control (Top) and Rab11aDN embryos at 2, 3, 4 and 7 days post fertilization (dpf).

**Supplemental Figure 3.**

**
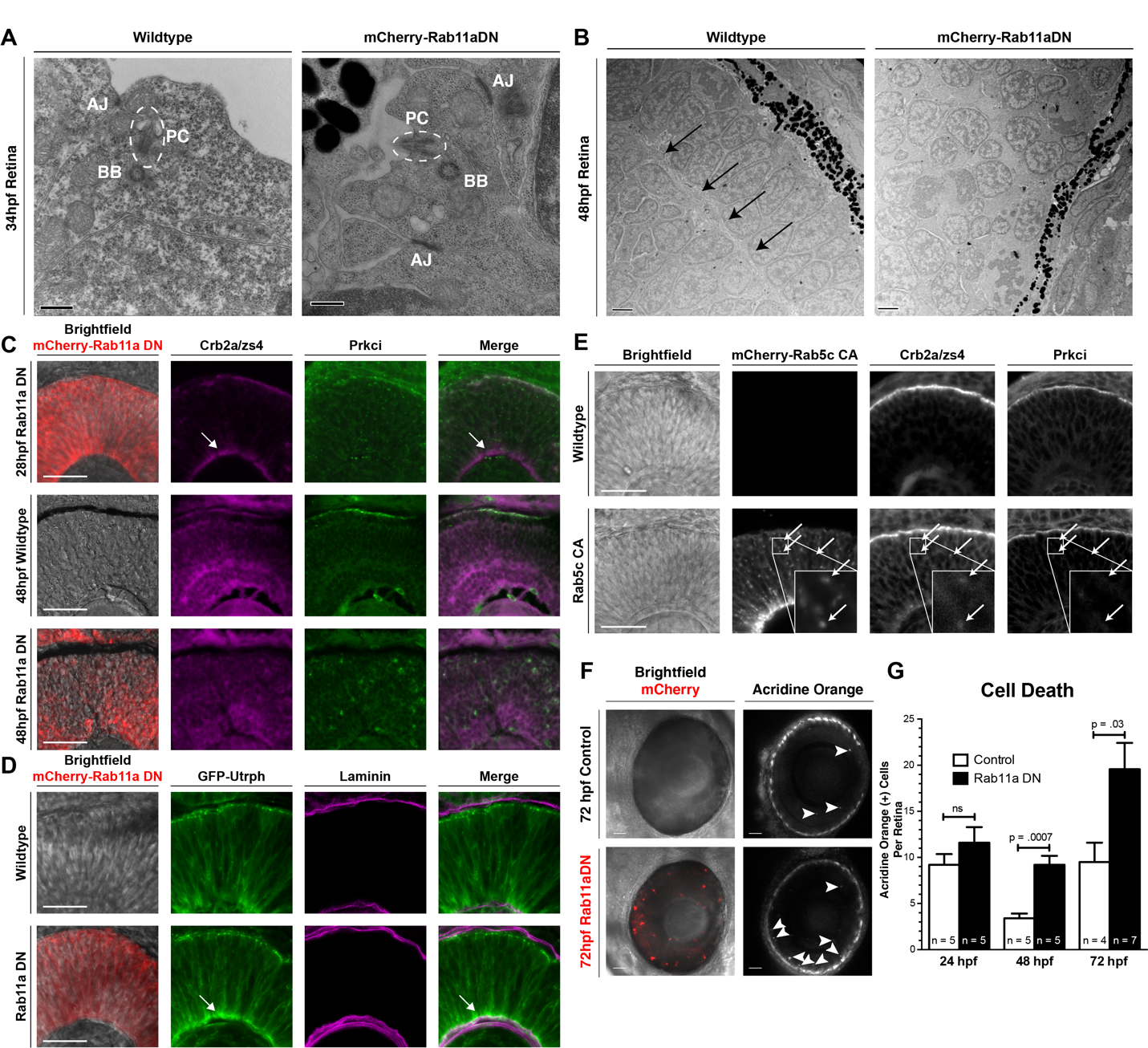
**

**Supplemental Figure 3. Consequences of Rab11aDN or Rab5cCA expression on retinal development. A)** Electron microscopy of 34hpf control (Wildtype; left) and Rabb1aDN RPCs indicating the presence of Apical Junctions (AJ), the Basal Body (BB) and Primary Cilia (PC) in both genotypes. **B)** Electron microscopy of 48hpf control (left) and Rab11aDN (right) retinas at the retina-RPE interface. Arrows indicate the beginnings of the outer plexiform layer within wildtype retinas. **C)** Immunostaining indicating Crb2a and Prkci localization in 28hpf Rab11aDN retinas (top) and 48hpf Control (middle) or Rabb11aDN retinas (bottom) **D)** Expression of apical actin-belt component Utrophin (EGFP-Utrph) and basement membrane component Laminin in Control (top) and Rab11aDN (bottom) 28hpf retinas. Arrows in C-D indicate basal accumulation of fluorescence. **E)** Expression of Crb2a and Prkci in 28hpf Control (top) and Rab5cCA (bottom) retinas. Arrows indicate localization of Rab5cCA endosomes containing Crb2a but lacking Prkci immunostaining. Despite the intracellular accumulation of Crb2a in Rab5cCA-positive early endosomes, a high degree of Crb2a fluorescence remains at the apical surface **F)** Representative examples of cells undergoing cell death (Acridine Orange-positive) in 72hpf Control (top) and Rab11aDN (bottom) retinas. **G)** Quantification of the average number dying cells as marked by Acridine Orange within control and Rab11aDN retinas at 24hpf, 48hpf, and 72hpf. Bar graphs in G represent categorical means, error bars represent SEM, and indicated p-values are the results of unpaired t-tests. Listed n-values represent number of embryos quantified. Scalebars in A represent 500nm. Scalebars in B represent 5µm. Scalebars in C- F represent 50µm

**Supplemental Figure 4.**

**
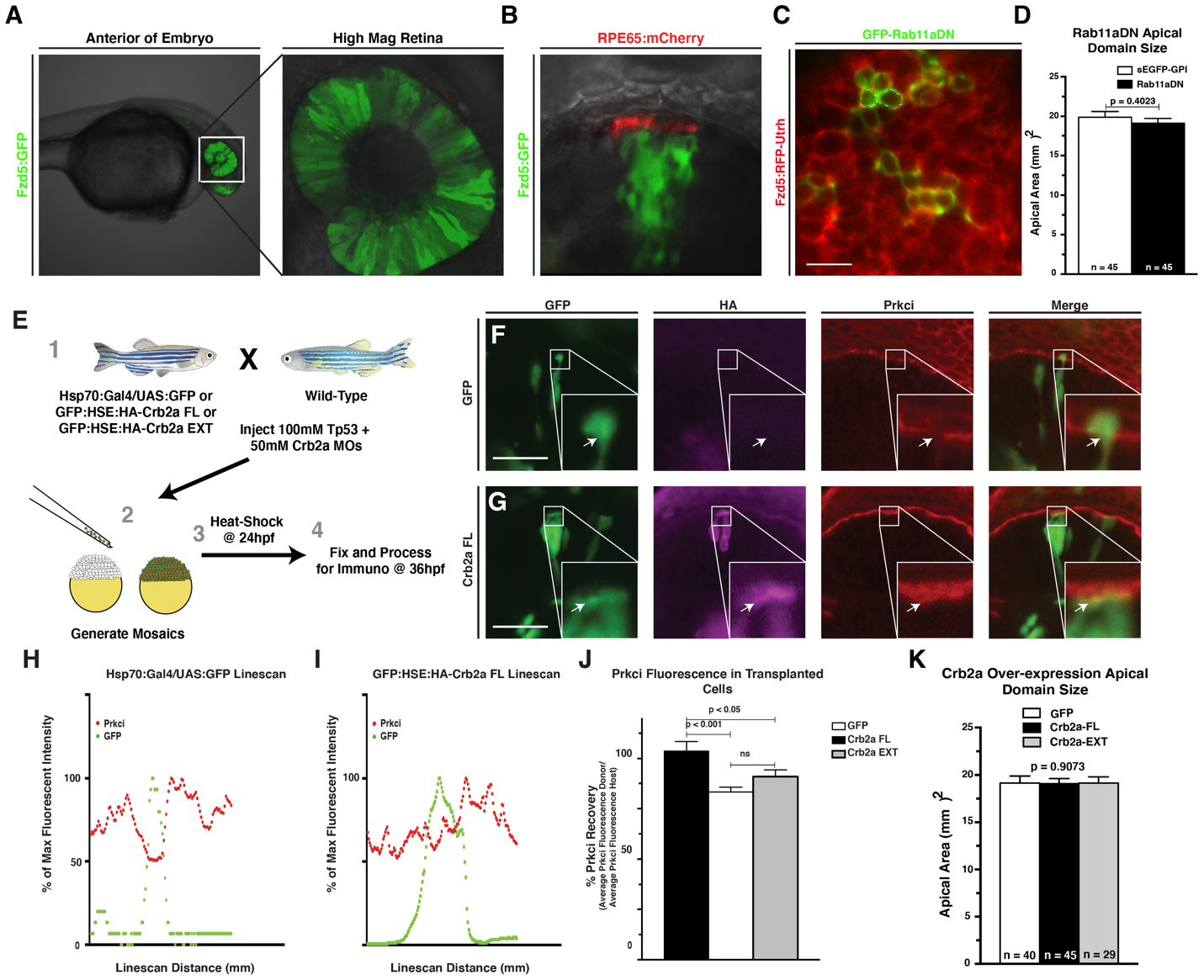
**

**Supplemental Figure 4. Expression of Rab11aDN or Crb2a transgenes does not affect apical domain size.** **A)** Characterization of the Fzd5 promoter driven expression of GFP specifically within the zebrafish RPCs **B)** Image indicating specificity of *Fzd5*:GFP and *Rpe65*:mCherry for the retina and RPE, respectively **C)** Example image examining the effect of Rab11aDN expression on RPC apical domain size as marked by retina-specific expression of RFP-Utrh. Outlines of GFP-Rab11aDN apical domains are indicated by the white dashed lines. **D)** Quantification of apical domain size in control (sEGFP-GPI) or Rab11aDN-expressing cells.

Graph represents mean apical domain size with SEM and statistics are the result of an unpaired t-test. **E)** Schematic of genetic mosaic experimental design assessing the Crb2a isoform dependence of Prkci localization within RPCs. **F-G)** Example clones within 36hpf retinas indicating Prkci localization in **F)** Control (Crb2a morphant; GFP expressing) cells or **G)** Crb2a-positive (Crb2a morphant; Crb2a-FL transgenic expression) cells. White boxes indicate regions of high-magnification insets. Arrows represent locations of apical domains of integrated clones **H-I)** Example line-scans of Prkci and GFP fluorescent intensities across apical membranes of **H)** Control (Crb2a morphant; GFP expressing) **I)** Crb2a-positive (Crb2a morphant; Crb2a-FL transgenic expression) cells **J)** Quantification of Prkci localization recovery in Crb2a-FL (Crb2a morphant; Crb2a-FL transgenic), Control (Crb2a morphant; GFP transgenic), and Crb2a-EXT (Crb2a morphant; Crb2a-EXT transgenic) cells as a percentage fluorescent intensity of average Prkci intensity of neighboring, wildtype (no Crb2a morpholino) cells. Results in J indicate mean percent recovery with error bars representing SEM. Listed p-values are the results of a Tukey’s multiple comparison test following a One-way ANOVA. **K)** Quantification of apical domain size in Control (*Hsp70:*GFP), Crb2a-FL or Crb2a-EXT expressing RPCs indicates no change in apical domain size resulting from Crb2a transgenic expression. Indicated n’s represent total number of cells quantified from >5 embryos/genotype. Graph indicates mean apical domain size with SEM with statistics representing the results of a One-way ANOVA. Scalebars in C indicate 10µm. Scalebars in F-G represent 50µm.

**Supplemental Figure 5.**

**
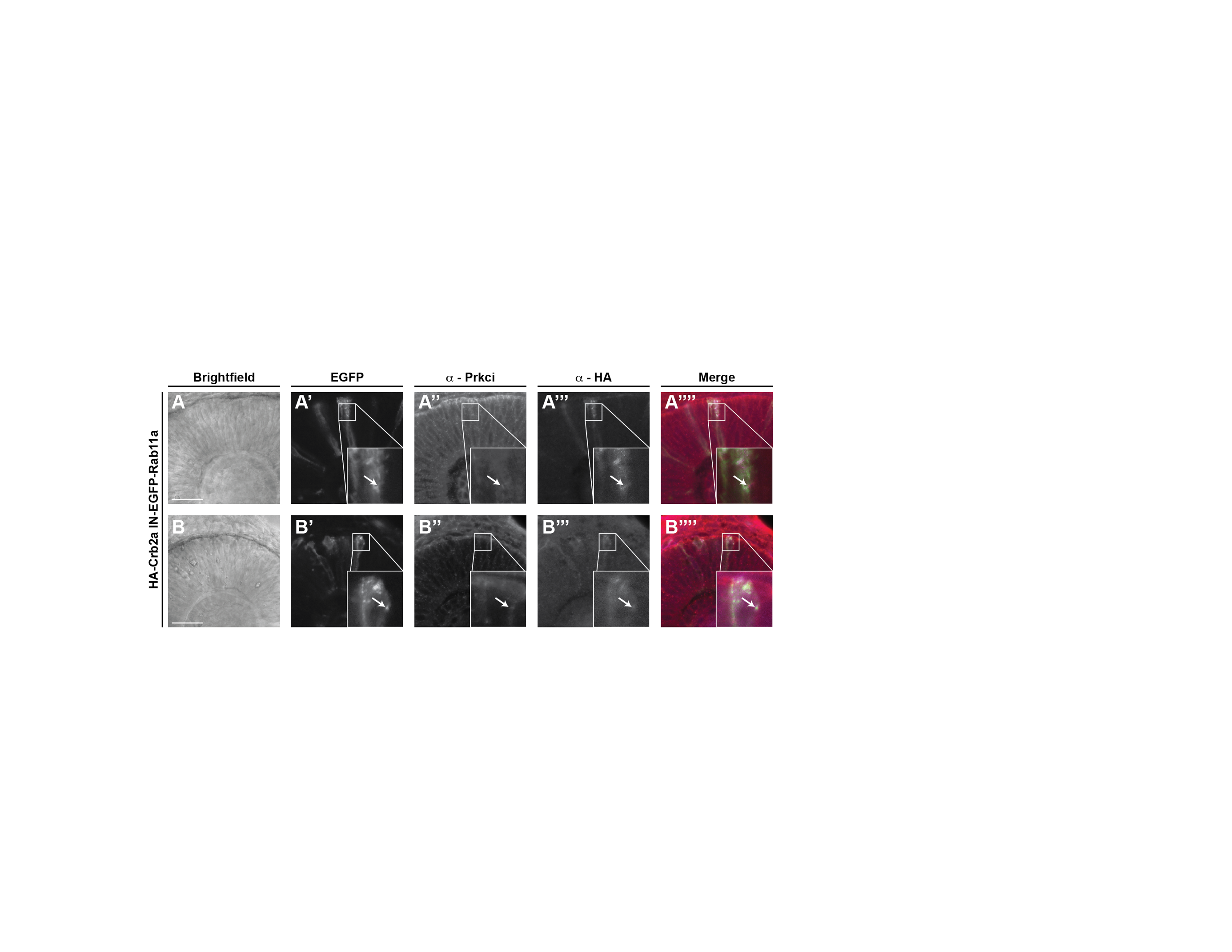
**

**Supplemental Figure 5. Forced expression of Crb2a IN to EGFP-Rab11a endosomes results in ectopic localization of Prkci. A-B)** Examples of recruitment of Prkci to sites of recycling endosome localized (EGFP-Rab11a; EGFP) Crb2a IN (HA)in 28hpf RPCs. White squares indicate regions of high magnification insets. Arrows indicate localization of intracellular puncta of Prkci across the fluorescent channels. Scalebars represent 50µm.

**Supplemental Figure 6.**

**
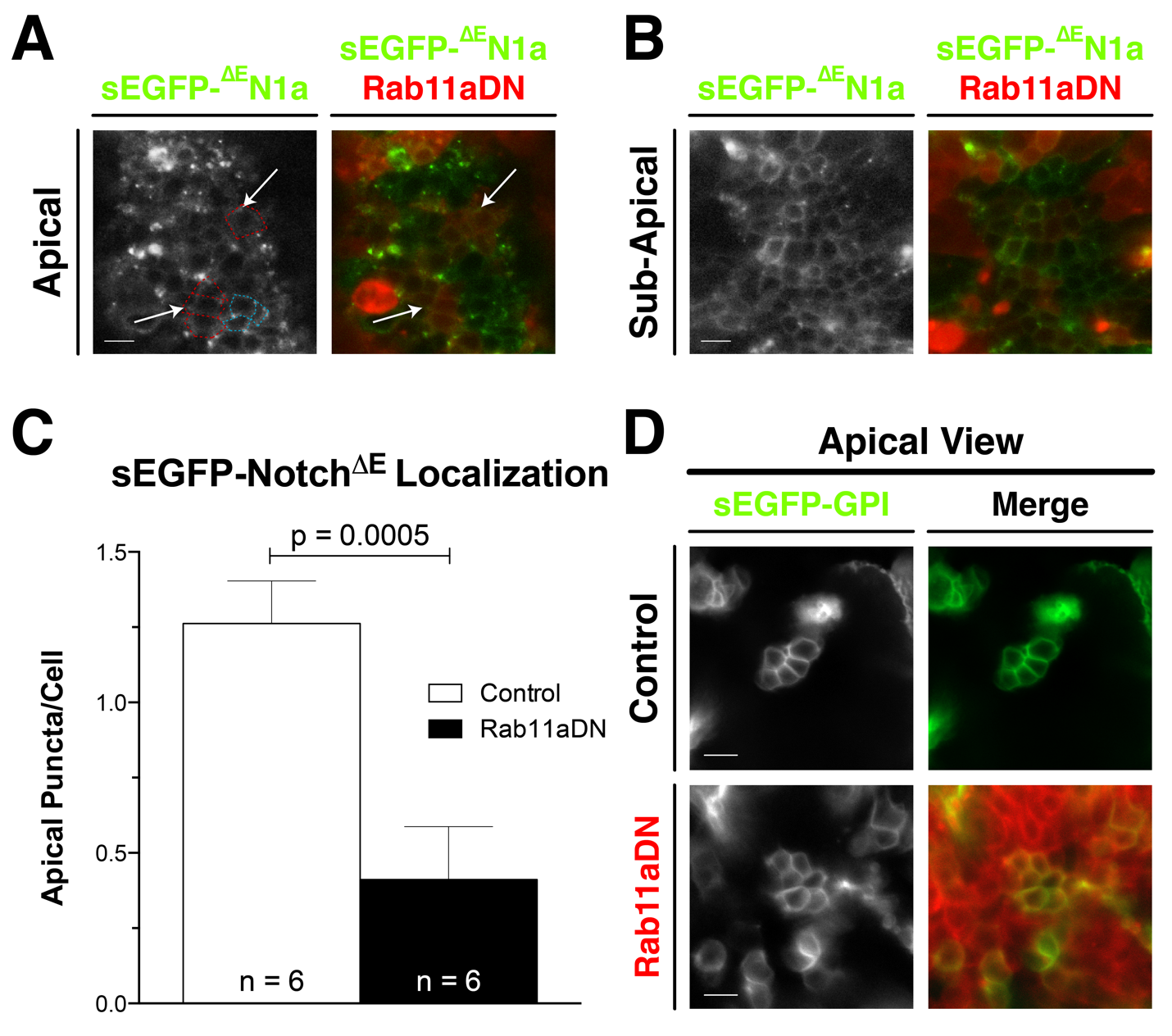
**

**Supplemental Figure 6. Rab11aDN expression prevents apical targeting of the sEGFP-Notch^ΔE^ transgene. A-B)** Representative images of the **A)** apical domain or **B)** sup-apical region of the sEGFP-Notch^ΔE^ transgene expression in Rab11aDN-positive cells. Arrows in A represent Rab11aDN-expressing cells devoid of apical sEGFP-NotchΔE transgene puncta. Regions outlined in red encompass regions quantified for large apical EGFP puncta. Regions outlined in blue indicate control cells. **C)** Quantification of apical sEGFP-Notch^ΔE^ within control or Rab11aDN-expressing RPCs. Results indicate mean apical EGFP puncta counted across >5 cells/retina, normalized to the number of cells counted, with error bars representing SEM. Listed n-values indicate number of retinas imaged and counted. Statistics represent results from an unpaired t-test **D)** Images of RPC apical domains assessing expression of a GPI-anchored secreted EGFP in Control (top panels) or Rab11aDN retinas, indicating generalized trafficking of proteins to the membrane is not affected by expression of the Rab11aDN transgene. Scalebars indicate 10µm.

**Supplemental Figure 7.**

**
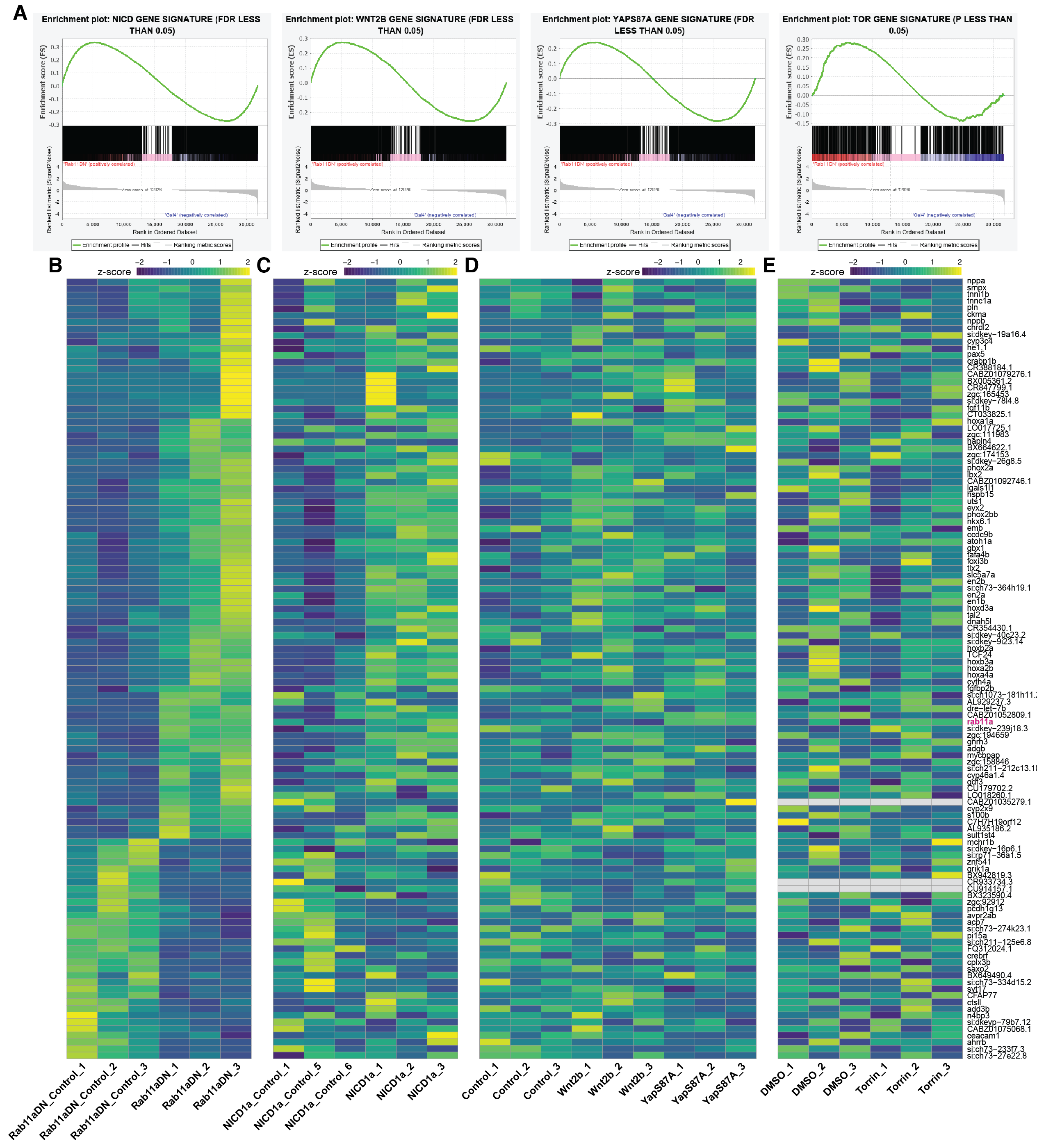
**

**Supplemental Figure 7. Comparisons of Rab11aDN differentially expressed transcripts across experimental paradigms. A)** Ingenuity pathway analysis assessing the ­­­­­overlap of differentially expressed transcripts from Rab11aDN experiments with Notch, Wnt2b, YapS87A and mTOR gene signatures **B)** Heatmaps of z-scores of top 117 differentially expressed transcripts (adjusted p-value < 0.05; log(|fold-change|) > 1.25) from Rab11aDN experiments across control and Rab11aDN replicates. **C-E)** Z-score expression signatures of top Rab11aDN differentially expressed transcripts across **C)** NICD1a **D)** Wnt2b and YapS87A, or **E)** Torin RNA-sequencing experiments.

**Supplemental Figure 8.**

**
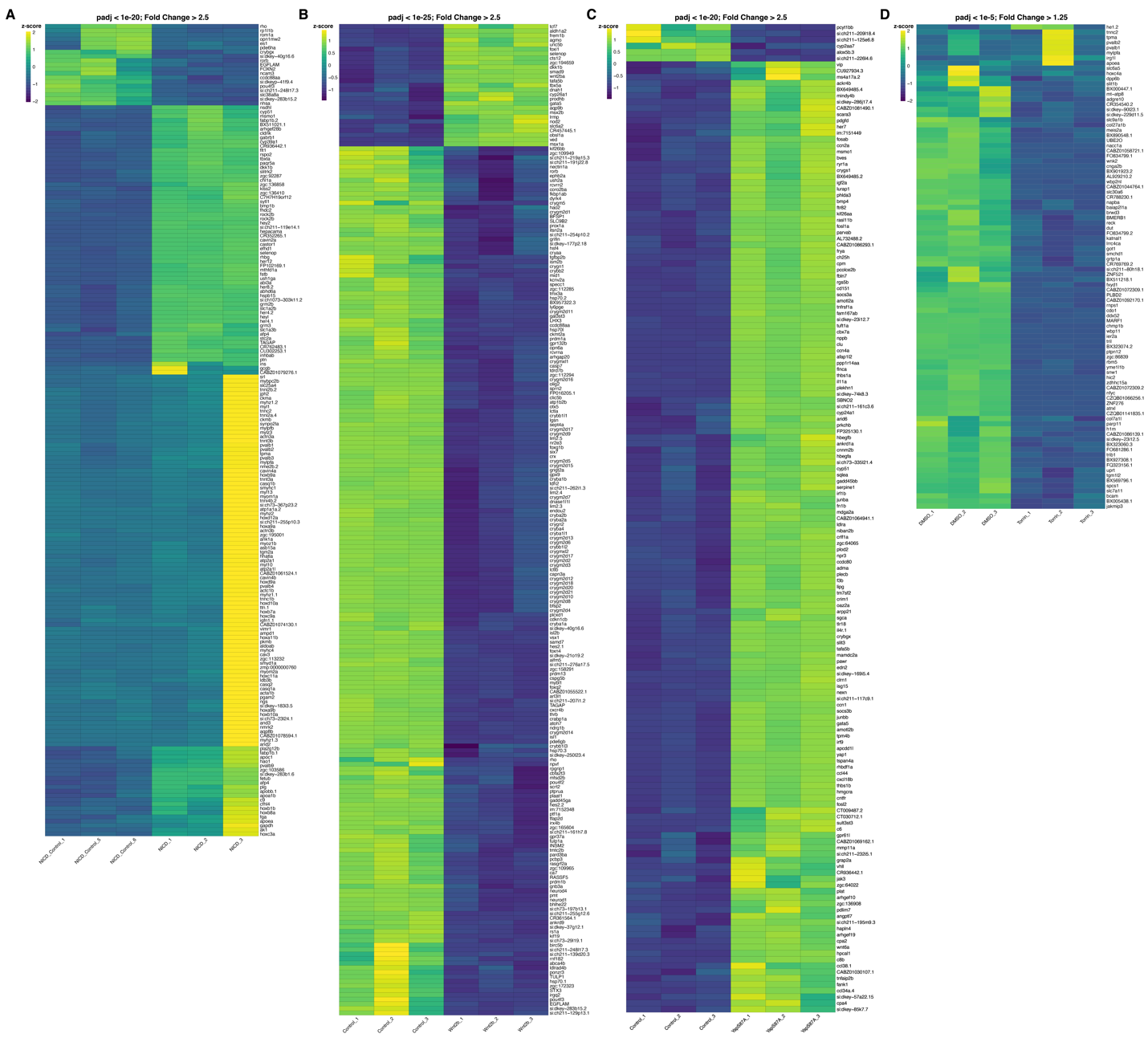
**

**Supplemental Figure 8. Differentially Gene Expression across RNA-sequencing experiments. A-D)** Z-scores of the top differentially expressed transcripts across biological replicates in **A)** NICD1a experiments (adjusted p-value < 1e-20; fold change >2.5) **B)** Wnt2b experiments (adjusted p-value < 1e-25; fold change > 2.5) **C)** YapS87A experiments (adjusted p-value < 1e-20; fold change > 2.5) or **D)** Torin experiments (adjusted p-value < 1e-5; fold change > 1.25)

**Supplemental Tables**

**Supplemental Table 1. RNA-sequencing QC Results.**

Table of total sequencing reads, including number of aligned reads and number of reads after read cleaning across each RNA-sequencing sample.

**Supplemental Table 2. edgeR Differential Expression Results.**

Results from pair-wise comparisons differential expression. Controls for Rab11aDN, NICD, Wnt2b, YapS87A, and Torrin experiments are Rab11aDN Control, NICD_Control, Control, Control, and DMSO, respectively.

**Supplemental Table 1. RNA-sequencing QC results**

**Supplemental Table 3. Transgenic and Mutant Alleles**

| **Transgenic and Mutant Alleles** | **Reference** |
| --- | --- |
| Tg(*h2afx:*EGFP-Rab5c)*^mw5^* | (Clark, Winter et al., 2011) |
| Tg(*h2afx*:EGFP-Rab11a)*^mw6^* | (Clark et al., 2011) |
| Tg(*h2afx*:EGFP-Rab7)*^mw7^* | (Clark et al., 2011) |
| Tg(*UAS*:mCherry-Rab11a S25N)*^mw35^* | (Clark et al., 2011) |
| Tg (*UAS*:mCherry-Rab5c Q81L)*^mw34^* | (Clark et al., 2011) |
| Tg (*vsx2*:Gal4vp16)*^mw39^* | (Clark et al., 2011) |
| Tg(*ßactin2*:man2a(1-100)-EGFP)*^mw4^* | This study; (Insinna, Baye et al., 2010) |
| Tg(*ef1alpha:*CoxVIII(35-127)-EGFP)*^mw19^* | This study; (Kim, Kang et al., 2008) |
| Tg(*ubi*:DsRED-ER)*^mw51^* | This study; cloned from pDsREd2-ER vector; Clontech (Mountain View, CA) Cat#632409 |
| Tg(*ßactin2*:mCherry-Golga2(806-1028))*^mw52^* | This study; (Barr, Nakamura et al., 1998) |
| Tg(*ßactin2*:EGFP-hCentrin)*^mw53^* | This study; (White, Doctor et al., 2000) |
| Tg(GFP:*HSE*:HA-Crb2a)*^mw54^* | This study; (Hsu & Jensen, 2010) |
| Tg(GFP:*HSE*:HA-Crb2a EXT (1-1430))*^mw55^* | This study; (Hsu et al. 2010) |
| Tg(*hfzd5*:GFP)*^mw56^* | This study; (Willardsen et al. 2009) |
| Tg(*hfzd5*:RFP-UtrCH)*^mw57^* | This study; (Burkel et al. 2007; Clark et. al., 2012;Willardsen et al., 2009) |
| Tg(*trß2*:EGFP)*^mw59^* | This study; (Suzuki, Bleckert et al., 2013) |
| Tg(*gnat2*:Gal4)*^mw60^* | This study; (Kennedy, Alvarez et al., 2007) |
| Tg(*UAS*:sEGFP-Notch1a**^Δ^**^E^)^mw93^ | This study; (Clark, Cui et al., 2012, Coffman, Skoglund et al., 1993) |
| Tg(*tp1-MmHbb*:dsGFP)*^mw43^* | (Clark et al., 2012) |
| Tg(*h2afx*:h2afv-mCherry)*^mw3^* | (McMahon, Gestri et al., 2009) |
| Tg(*her4*:dRED)*^knu2^* | (Yeo, Kim et al., 2007) |
| Tg(*atoh7*:GFP)*^rw021^* | (Masai, Lele et al., 2003) |
| Tg(*h2afv*:h2afv-GFP)*^kca6^* | (Pauls, Geldmacher-Voss et al., 2001) |
| Tg(*hsp70l*:GFP) | (Halloran, Sato-Maeda et al., 2000) |
| Tg(-1.5 *hsp70*:Gal4)*^ka4^* | (Scheer, Riedl et al., 2002) |
| Tg(*5x UAS*:GFP)*^zf82^* | (Asakawa & Kawakami, 2008) |
| *ome* (*crb2a)* | (Malicki & Driever, 1999) |
| Tg(*UAS*:myc-Notch1a-ICD) | (Scheer & Campos-Ortega, 1999) |
| Tg(*UAS*:EGFP-Wnt2ba) ^mw94^ | This study |
| Tg(dsRED:*UAS*:YapS87A) ^mw65^ | (Miesfeld, Gestri et al., 2015) |

**Supplemental Table 4**

| **Constructs generate for use in Clark *et al,* Rab11aDN and neurogenesis** |  |
| --- | --- |
| Constructs are available upon request |  |
|  |  |
| **Entry Clones (3-Way Tol2 Gateway System)** | **Notes** |
| pME-man2a(1-100) |  |
| p3E-hCentrin | Human centrin cDNA |
| pME-Cox VIII (35-127) |  |
| p3E-Golga2(806-1028) | GM130 |
| pME-DsRED-ER | ER targeting and KDEL ER retention sequences fused to DsRED |
| p3E-Rab11a |  |
| p3E-Rab11a S25N | Rab11a Dominant Negative |
| p5E-*hFzd5* | from the pG1-cfos-hFzd5CSA:GFP construct (gift from M. Vetter, University of Utah), see methods (Willardsen, Suli et al., 2009)^[[1]](#endnote-1)^ |
| p3E-UtrCH |  |
| pME-sEGFP | secreted GFP; remains in frame with C-terminal fusions |
| p5E-*trb2* | see methods (Suzuki, 2013a) |
| p5E-*gnat2* | 3169bp of gnat2 proximal promoter |
| p3E-myc Notch1aDE |  |
| p3E-Wnt2ba |  |
| pME-HA-Crb2a FL | Gift from Abbie Jensen; HA inserted downstream of the endogenous Crb2a signal peptide |
| pME-HA-Crb2a EXT | Gift from Abbie Jensen; HA inserted downstream of the endogenous Crb2a signal peptide |
| pME-HA-Crb2a INT | Gift from Abbie Jensen; HA inserted downstream of the endogenous Crb2a signal peptide |
| pME-HA-Crb2aIN EGFP | Lawson Lab clone #443; HA-Crb2aIN inserted into the NcoI site 5' to EGFP; direct fusion of HA to Crb2aIN |
| p5E-nlsEGFP:*8X HSE*: | nls sequence inserted upstream of EGFP of p5E-EGFP:8X HSE: |
| p5E-EGFP:*8X HSE*: | generated from psGH containing 8xHSE bidirectional gfp polylinker (Bajoghli, Aghaallaei et al., 2004) |
| p5E-RPE65 | 818bp of RPE65 proximal promoter |
|  |  |
| **Tol2 Constructs** |  |
| *Tol2 - b-actin2:*man2a(1-100)-EGFP |  |
| *Tol2 - ef1a:*Cox VIII (35-127)-EGFP |  |
| *Tol2 - b-actin2:*mCherry-Golga2(806-1028) |  |
| *Tol2 - ubi:*DsRED-ER |  |
| *Tol2 - b-actin2:*EGFP-hCentrin |  |
| *Tol2 -* EGFP:*8X HSE*:HA-Crb2a FL |  |
| *Tol2 -* EGFP:*8X HSE*:HA-Crb2a EXT |  |
| *Tol2 -* EGFP:*8X HSE*:HA-Crb2a INT |  |
| *Tol2 -* nlsEGFP*:8X HSE:*HA-Crb2a FL |  |
| *Tol2 -* nlsEGFP*:8X HSE:*HA-Crb2a EXT |  |
| *Tol2 -* nlsEGFP*:8X HSE:*HA-Crb2a INT |  |
| *Tol2 -* nlsEGFP*:8X HSE*:HA-Crb2a IN EGFP-Rab11a |  |
| *Tol2 -* nlsEGFP*:8X HSE*:EGFP-Rab11a |  |
| *Tol2 -* nlsEGFP*:8X HSE*:H2a-mCherry |  |
| *Tol2* - *hsp70*:mCherry-Rab11a S25N |  |
| *Tol2* - *hsp70*:EGFP-Rab11a S25N |  |
| *Tol2* - *UAS*:mCherry-Rab11a S25N |  |
| *Tol2* - *UAS*:EGFP-Rab11a S25N |  |
| *Tol2 - hFzd5*:GFP |  |
| *Tol2 - hFzd5*:RFP-UtrCH |  |
| *Tol2* - *UAS*:sEGFP-mycNotch1aDE |  |
| *Tol2 - RPE65*:mcherry |  |
| *Tol2 –* dsRed*:UAS:*Evi5b |  |

**Supplemental References**

Asakawa K, Kawakami K (2008) Targeted gene expression by the Gal4-UAS system in zebrafish. *Dev Growth Differ* 50: 391-9

Bajoghli B, Aghaallaei N, Heimbucher T, Czerny T (2004) An artificial promoter construct for heat-inducible misexpression during fish embryogenesis. *Dev Biol* 271: 416-30

Barr FA, Nakamura N, Warren G (1998) Mapping the interaction between GRASP65 and GM130, components of a protein complex involved in the stacking of Golgi cisternae. *EMBO J* 17: 3258-68

Clark BS, Cui S, Miesfeld JB, Klezovitch O, Vasioukhin V, Link BA (2012) Loss of Llgl1 in retinal neuroepithelia reveals links between apical domain size, Notch activity and neurogenesis. *Development* 139: 1599-610

Clark BS, Winter M, Cohen AR, Link BA (2011) Generation of Rab-based transgenic lines for in vivo studies of endosome biology in zebrafish. *Dev Dyn* 240: 2452-65

Coffman CR, Skoglund P, Harris WA, Kintner CR (1993) Expression of an extracellular deletion of Xotch diverts cell fate in Xenopus embryos. *Cell* 73: 659-71

Halloran MC, Sato-Maeda M, Warren JT, Su F, Lele Z, Krone PH, Kuwada JY, Shoji W (2000) Laser-induced gene expression in specific cells of transgenic zebrafish. *Development* 127: 1953-60

Hsu YC, Jensen AM (2010) Multiple domains in the Crumbs Homolog 2a (Crb2a) protein are required for regulating rod photoreceptor size. *BMC Cell Biol* 11: 60

Insinna C, Baye LM, Amsterdam A, Besharse JC, Link BA (2010) Analysis of a zebrafish dync1h1 mutant reveals multiple functions for cytoplasmic dynein 1 during retinal photoreceptor development. *Neural Dev* 5: 12

Kennedy BN, Alvarez Y, Brockerhoff SE, Stearns GW, Sapetto-Rebow B, Taylor MR, Hurley JB (2007) Identification of a zebrafish cone photoreceptor-specific promoter and genetic rescue of achromatopsia in the nof mutant. *Invest Ophthalmol Vis Sci* 48: 522-9

Kim MJ, Kang KH, Kim CH, Choi SY (2008) Real-time imaging of mitochondria in transgenic zebrafish expressing mitochondrially targeted GFP. *Biotechniques* 45: 331-4

Malicki J, Driever W (1999) *oko meduzy* mutations affect neuronal patterning in the zebrafish retina and reveal cell-cell interactions of the retinal neuroepithelial sheet. *Development* 126: 1235-46

Masai I, Lele Z, Yamaguchi M, Komori A, Nakata A, Nishiwaki Y, Wada H, Tanaka H, Nojima Y, Hammerschmidt M, Wilson SW, Okamoto H (2003) N-cadherin mediates retinal lamination, maintenance of forebrain compartments and patterning of retinal neurites. *Development* 130: 2479-94

McMahon C, Gestri G, Wilson SW, Link BA (2009) Lmx1b is essential for survival of periocular mesenchymal cells and influences Fgf-mediated retinal patterning in zebrafish. *Dev Biol* 332: 287-98

Miesfeld JB, Gestri G, Clark BS, Flinn MA, Poole RJ, Bader JR, Besharse JC, Wilson SW, Link BA (2015) Yap and Taz regulate retinal pigment epithelial cell fate. *Development* 142: 3021-32

Pauls S, Geldmacher-Voss B, Campos-Ortega JA (2001) A zebrafish histone variant H2A.F/Z and a transgenic H2A.F/Z:GFP fusion protein for in vivo studies of embryonic development. *Dev Genes Evol* 211: 603-10

Scheer N, Campos-Ortega JA (1999) Use of the Gal4-UAS technique for targeted gene expression in the zebrafish. *Mech Dev* 80: 153-8

Scheer N, Riedl I, Warren JT, Kuwada JY, Campos-Ortega JA (2002) A quantitative analysis of the kinetics of Gal4 activator and effector gene expression in the zebrafish. *Mech Dev* 112: 9-14

Suzuki SC, Bleckert A, Williams PR, Takechi M, Kawamura S, Wong RO (2013) Cone photoreceptor types in zebrafish are generated by symmetric terminal divisions of dedicated precursors. *Proc Natl Acad Sci U S A* 110: 15109-14

White P, Doctor RB, Dahl RH, Chen J (2000) Coincident microvillar actin bundle disruption and perinuclear actin sequestration in anoxic proximal tubule. *Am J Physiol Renal Physiol* 278: F886-93

Willardsen MI, Suli A, Pan Y, Marsh-Armstrong N, Chien CB, El-Hodiri H, Brown NL, Moore KB, Vetter ML (2009) Temporal regulation of Ath5 gene expression during eye development. *Dev Biol* 326: 471-81

Yeo SY, Kim M, Kim HS, Huh TL, Chitnis AB (2007) Fluorescent protein expression driven by her4 regulatory elements reveals the spatiotemporal pattern of Notch signaling in the nervous system of zebrafish embryos. *Dev Biol* 301: 555-67

1. [↑](#endnote-ref-1)
